# Artificial Intelligence in Nanopharmaceutical Development: From Predictive Design to Clinical Translation

**DOI:** 10.3390/pharmaceutics18060764

**Published:** 2026-06-22

**Authors:** Renato Sonchini Gonçalves

**Affiliations:** Department of Engineering and Exact Sciences, Palotina Sector, Federal University of Paraná (UFPR), Palotina 85950-000, PR, Brazil; renato.sonchini@ufpr.br; Tel.: +55-98-985-149-235

**Keywords:** machine learning, deep learning, drug delivery, nano–bio interactions, quality-by-design, process analytical technology, digital twins, pharmacokinetics, toxicity prediction, regulatory science

## Abstract

Artificial intelligence (AI) is increasingly influencing nanopharmaceutical development by supporting the transition from empirical formulation screening toward predictive, data-driven, and translationally oriented design. Nanocarrier-based therapeutics are governed by nonlinear relationships among material composition, physicochemical attributes, manufacturing parameters, biological identity, pharmacokinetics, toxicity, and therapeutic performance. In this review, we examine how AI can contribute to nanopharmaceutical development from predictive formulation design to clinical translation. We synthesize current applications of machine learning, deep learning, physics-informed modeling, hybrid mechanistic–AI approaches, and automated optimization workflows, with emphasis on critical quality attribute modeling, multi-objective optimization, design of experiments, quality-by-design, process analytical technology, digital twins, and continuous manufacturing. We also discuss applications involving nano–bio interactions, pharmacokinetics, toxicity, immunogenicity, and precision nanomedicine. AI-based approaches can support rational nanocarrier design, identify nonlinear formulation–property relationships, guide optimization, improve process understanding, and integrate heterogeneous experimental, biological, and manufacturing datasets across diverse nanopharmaceutical platforms. These methods are particularly relevant for modeling protein corona formation, cellular uptake, intracellular trafficking, biodistribution, pharmacokinetics, toxicity, immunogenicity, and patient-specific responses. However, translational implementation remains limited by fragmented datasets, inconsistent reporting standards, limited interpretability, insufficient external validation, uncertain predictions, poorly defined applicability domains, and evolving regulatory expectations for adaptive computational models. Overall, AI should be viewed not only as an optimization tool, but also as a translational framework connecting formulation science, biological prediction, manufacturing control, and clinical implementation. Future progress will depend on standardized data infrastructures, explainable and externally validated models, uncertainty quantification, applicability-domain definition, hybrid mechanistic–AI frameworks, regulatory-ready documentation, and clinically relevant case studies.

## 1. Introduction

Nanopharmaceuticals occupy a distinctive position in advanced drug delivery because their therapeutic performance depends not only on the pharmacological activity of the drug, but also on the engineered properties of the carrier. Nanoscale systems can improve solubility, bioavailability, biodistribution, controlled release, and site-specific delivery, but these advantages are closely linked to formulation-dependent attributes such as particle size, surface charge, morphology, composition, drug loading, surface functionalization, and stimuli-responsive behavior [1,2,3,4]. As a result, nanopharmaceutical development requires simultaneous consideration of material design, manufacturing reproducibility, biological interactions, safety, and clinical feasibility. This multidimensional complexity is precisely what makes nanomedicine scientifically attractive, but also difficult to translate reliably from experimental models to approved therapies.

Despite these advantages, the clinical trajectory of nanopharmaceuticals has been more difficult than early expectations suggested. Several nanoparticle-based products have reached the clinic, but the number of approved systems remains small compared with the large and expanding preclinical literature [5,6,7]. This gap is not explained by carrier design alone. It reflects a broader set of translational obstacles, including batch-to-batch reproducibility, manufacturing scale-up, biological variability, safety assessment, regulatory evidence requirements, and the limited ability of preclinical models to predict human performance [6,7,8,9]. In practice, small changes in particle size distribution, surface chemistry, composition, or processing conditions can alter colloidal stability, drug release, protein corona formation, biodistribution, clearance, toxicity, and therapeutic response [4,7,9]. Therefore, the main challenge is not simply to design nanoparticles with desirable physicochemical properties, but to understand which formulation and process variables are most likely to produce reproducible biological and clinical benefit. Together, these issues define the translational bottleneck addressed in this review: the need to predict how formulation and manufacturing variables influence biological performance and clinical feasibility before extensive late-stage experimentation.

Structured development frameworks have made formulation development more systematic, but they have not fully solved the predictive problem in nanopharmaceutical design. Design of experiments and quality-by-design provide useful tools for mapping formulation variables, defining critical material attributes, identifying critical process parameters, and linking them to critical quality attributes [10,11]. Process analytical technology, referring to real-time or near-real-time monitoring tools used to evaluate and control manufacturing processes, further supports a science- and risk-based approach to pharmaceutical development, manufacturing, and quality assurance [12]. However, these approaches are often most informative within predefined experimental spaces. Their limitations become more evident when nanopharmaceutical datasets are high-dimensional, heterogeneous, nonlinear, or generated across different experimental, biological, analytical, and manufacturing contexts. The increasing availability of imaging, omics, pharmacokinetic, high-throughput screening, and process analytical technology data therefore creates a need for computational frameworks capable of integrating complex data streams. In this context, nanopharmaceutical innovation increasingly requires approaches that connect formulation science, manufacturing information, and biological performance within a single predictive framework [10,11,12].

Artificial intelligence offers a way to address this predictive gap by learning patterns that are difficult to capture with conventional empirical or statistical models. Machine learning, deep learning, physics-informed modeling, and hybrid mechanistic–AI strategies, defined here as approaches that combine data-driven models with mechanistic knowledge of formulation, transport, release, or biological processes, can identify relationships among formulation variables, manufacturing parameters, physicochemical attributes, and biological outcomes [13,14,15]. Compared with conventional empirical or response-surface approaches, AI-based models can handle nonlinear and multidimensional formulation–performance relationships with greater flexibility [13,14]. Recent studies on AI in drug delivery and nanomedicine have highlighted applications in formulation optimization, critical parameter prediction, material selection, and biological response modeling, while also emphasizing that reliable AI use depends on dataset quality, descriptor relevance, algorithm selection, validation, and interpretability [13,14,15,16].

Nevertheless, the application of AI in nanopharmaceutical development must be interpreted critically. Many current studies remain proof-of-concept analyses based on small, heterogeneous, or insufficiently standardized datasets [13,14,15,16]. Models trained under narrow experimental conditions may perform well during internal validation but fail when applied to different nanocarrier types, biological models, laboratories, or manufacturing scales. Therefore, the translational value of AI depends not only on algorithmic sophistication, but also on data quality, descriptor standardization, external validation, uncertainty quantification, interpretability, and integration with domain knowledge [13,15,16]. Despite these limitations, practical examples already demonstrate the potential of AI in nanopharmaceutical research. For instance, machine learning models have been used to predict formulation attributes of poly(lactic-co-glycolic acid) nanoparticles, including particle size, polydispersity, drug loading, and encapsulation efficiency, while AI-assisted physiologically based pharmacokinetic modeling has been applied to predict nanoparticle delivery and biodistribution [17,18].

The value of AI in this field is greatest when it is embedded within established pharmaceutical development frameworks rather than treated as an isolated computational layer. When combined with design of experiments, quality-by-design, process analytical technology, and digital twins, defined as virtual representations of physical systems that are updated with experimental or process data, AI can support adaptive experimental design, closed-loop optimization, real-time process monitoring, and continuous model refinement [10,11,12,19]. These applications are particularly relevant for nanopharmaceuticals because formulation performance depends on variables that are difficult to control independently, including material composition, process conditions, biological identity, and manufacturing scale. However, AI-supported systems also introduce new responsibilities. Models used for process monitoring, quality decisions, or formulation selection require clear objectives, traceable data, validation strategies, lifecycle management, and documentation of model updates [12,16,19]. They also raise ethical, privacy, and societal considerations when patient-specific data, adaptive decision-support tools, or precision nanomedicine strategies are involved. These issues are discussed later in relation to clinical translation, governance, and responsible implementation [16,20].

A second area in which AI may add value is the prediction of nano–bio interactions, which remain one of the main sources of translational uncertainty in nanopharmaceutical development. After administration, nanocarriers are rapidly transformed by their biological environment through protein corona formation, cellular uptake, intracellular trafficking, immune recognition, tissue accumulation, clearance, and drug release [4,21,22]. These processes cannot be explained by physicochemical descriptors alone, because the same nanocarrier may behave differently depending on biological medium, cell type, disease state, administration route, immune status, and patient-specific factors. AI-based models can help address this complexity by integrating physicochemical descriptors with proteomic, imaging, toxicological, pharmacokinetic, and clinical datasets [13,21,22]. However, such models require careful validation because biological datasets are highly context-dependent and may not transfer across experimental models, laboratories, animal species, or patient populations [7,9,21].

This review examines artificial intelligence as a translational framework for nanopharmaceutical development rather than as a collection of isolated computational tools. The focus is on how AI can support predictive formulation design, advanced modeling, data-driven optimization, nano–bio interaction analysis, pharmacokinetic prediction, toxicity assessment, scalable manufacturing, and regulatory decision-making. Compared with reviews that broadly describe AI applications in drug delivery or nanomedicine, this work emphasizes the conditions under which AI may become practically useful: biologically meaningful descriptors, standardized datasets, interpretable models, uncertainty-aware predictions, external validation, and evidence that computational outputs improve formulation, manufacturing, or translational decisions.

This review emphasizes four main points. First, nanopharmaceutical translation remains limited by the difficulty of predicting relationships among formulation composition, manufacturing parameters, physicochemical attributes, and biological responses. Second, AI-based approaches can support predictive formulation design, optimization, nano–bio interaction modeling, pharmacokinetic prediction, safety assessment, and manufacturing control. Third, the translational value of AI depends on data quality, descriptor standardization, interpretability, external validation, uncertainty quantification, and regulatory-ready documentation. Finally, AI should be considered a complementary framework that extends, rather than replaces, established pharmaceutical development approaches such as design of experiments, quality-by-design, process analytical technology, and mechanistic modeling.

**Figure 1:**
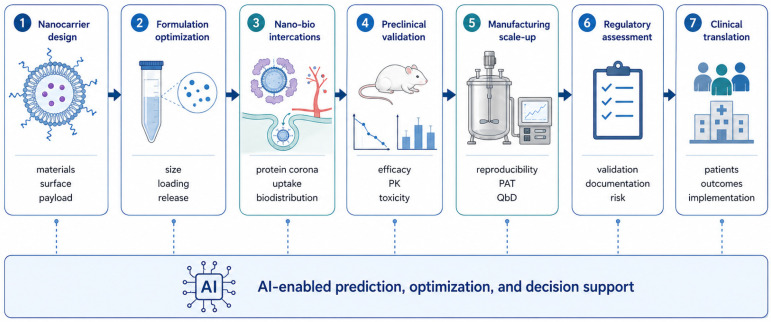
AI-supported nanopharmaceutical development from design to clinical translation. The workflow summarizes the main stages of nanopharmaceutical development, including nanocarrier design, formulation optimization, nano–bio interaction analysis, preclinical validation, manufacturing scale-up, regulatory assessment, and clinical translation. Across these stages, AI can support prediction, optimization, and decision-making by integrating formulation, biological, manufacturing, and translational data.

To orient the reader, Figure 1 provides an integrated overview of the AI-driven nanopharmaceutical development pipeline, linking nanocarrier design, formulation optimization, nano–bio interaction modeling, clinical translation, and regulatory implementation.

## 2. Advanced Modeling Approaches for Nanopharmaceutical Design

The rational design of nanopharmaceutical systems remains challenging because formulation performance is governed by nonlinear and interdependent relationships among material composition, physicochemical attributes, processing conditions, and biological responses [23,24]. Parameters such as particle size, polydispersity, surface charge, drug loading, release kinetics, colloidal stability, and targeting efficiency cannot be interpreted as isolated variables, since changes in one attribute may substantially affect cellular uptake, biodistribution, clearance, toxicity, and therapeutic efficacy [23,24,25]. This complexity limits the predictive value of conventional empirical strategies and supports the need for more integrated computational approaches.

Traditional formulation development has relied on trial-and-error experimentation and, more recently, on statistical tools such as response surface methodology and design of experiments. Although these approaches provide structure and improve the identification of critical formulation and process variables, they are generally more effective within predefined experimental spaces and may be limited when datasets are high-dimensional, heterogeneous, nonlinear, or derived from multiple analytical and biological sources [26,27]. Compared with these classical approaches, artificial intelligence, including machine learning, deep learning, and hybrid modeling strategies, offers greater flexibility for moving from descriptive optimization toward predictive formulation design [23,25,28].

AI-based approaches are particularly relevant for nanopharmaceutical systems because they can capture nonlinear interactions, process large descriptor spaces, and integrate multimodal datasets from physicochemical characterization, high-throughput screening, imaging, omics analyses, and pharmacokinetic studies [23,24,28]. Machine learning models can support the prediction of critical quality attributes such as particle size, zeta potential, encapsulation efficiency, drug loading, stability, and release behavior [17,23]. Machine learning models can support the prediction of critical quality attributes such as particle size, polydispersity, zeta potential, encapsulation efficiency, drug loading, stability, and release behavior [17,23,29,30,31]. These applications illustrate how AI can transform formulation datasets into predictive tools for nanocarrier design, while also emphasizing that model generalizability depends on dataset diversity, descriptor standardization, and validation across different nanocarrier platforms [23,30,31].

Deep learning methods, in turn, can extract complex information from unstructured data, including microscopy images, spectral profiles, and high-content biological assays [24,28]. Compared with conventional machine learning, deep learning is better suited for image-rich and multimodal datasets, but it generally requires larger, well-annotated datasets and stronger interpretability strategies. These capabilities make AI useful not only for accelerating formulation screening but also for identifying hidden formulation drivers and prioritizing experimentally relevant design regions.

However, AI-driven modeling must be interpreted critically. High predictive accuracy during internal validation does not necessarily indicate translational robustness, especially when models are trained on small, biased, or poorly standardized datasets [23,24,28]. Many published models remain limited by insufficient external validation, inconsistent reporting of nanoparticle descriptors, and limited reproducibility across laboratories. Therefore, the scientific value of AI in nanopharmaceutical design depends not only on algorithmic performance but also on data quality, descriptor relevance, model interpretability, biological plausibility, and experimental validation [23,25,28].

**Figure 2:**
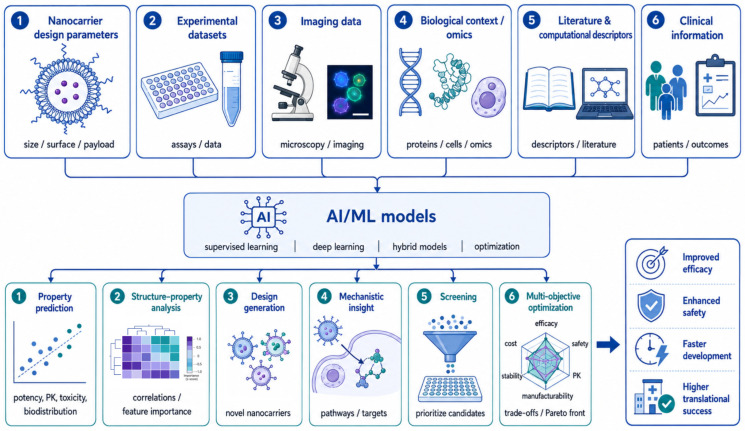
Artificial intelligence/machine learning (AI/ML)-driven workflow for nanopharmaceutical design and optimization. The figure illustrates how nanocarrier design parameters, experimental datasets, imaging data, biological context, literature-derived descriptors, and clinical information can be integrated into AI/ML models. These models support property prediction, structure–property analysis, design generation, mechanistic insight, candidate screening, and multi-objective optimization, contributing to improved efficacy, enhanced safety, faster development, and higher translational success.

As illustrated in Figure 2, the integration of AI into nanopharmaceutical development should be understood as a structured and iterative pipeline involving data acquisition, preprocessing, feature engineering, model selection, training, validation, interpretation, and experimental feedback. In this workflow, feature engineering is particularly important because model performance depends strongly on the quality and biological relevance of input descriptors [23,25]. Variables related to nanoparticle composition, size distribution, surface chemistry, preparation method, stabilizer type, drug properties, and experimental conditions must therefore be curated and standardized to support reliable model development.

A particularly important direction is the combination of data-driven models with mechanistic and physics-informed approaches. Purely data-driven models may provide strong predictive performance but often lack interpretability and extrapolation capacity, especially when predictions are extended beyond the experimental domain represented in the training dataset [32,33]. Hybrid models, by contrast, combine machine learning with mechanistic knowledge of diffusion, dissolution, transport phenomena, colloidal stability, and pharmacokinetics. Compared with purely empirical models, these approaches can improve generalizability, reduce data requirements, and generate predictions that are more scientifically defensible, particularly for regulatory and translational applications [32,33,34]. This is especially relevant in nanopharmaceutical development, where extrapolation across formulation types, biological environments, or manufacturing scales is often required but rarely supported by data-driven models alone [33,34].

**Figure 3:**
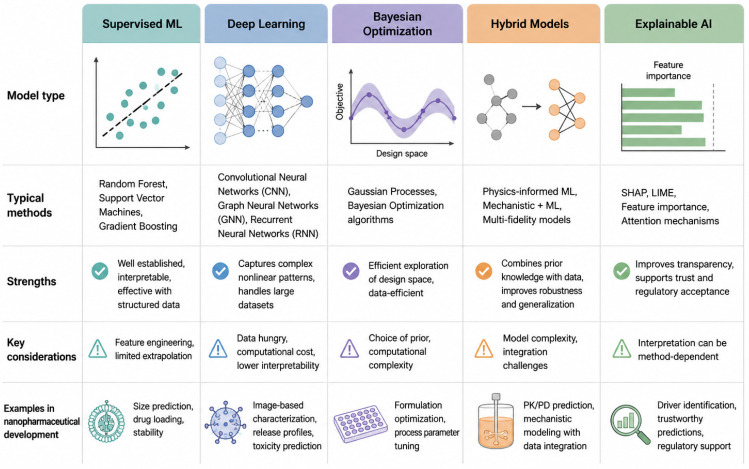
Comparative overview of AI modeling strategies for nanopharmaceutical development. The figure compares representative AI approaches, including supervised machine learning, deep learning, Bayesian optimization, hybrid models, and explainable AI. Each approach differs in typical methods, strengths, limitations, and suitability for nanopharmaceutical applications such as formulation–property prediction, image-based characterization, optimization, pharmacokinetic modeling, and regulatory-oriented interpretation.

Overall, advanced modeling approaches are reshaping nanopharmaceutical design by enabling a transition from empirical formulation screening to predictive, interpretable, and optimization-driven development [23,24,25,32]. However, their successful implementation depends not only on algorithmic sophistication, but also on standardized datasets, rigorous validation, explainable models, and integration with experimental workflows [24,28,33,35]. To provide an integrated overview of the computational strategies discussed in the following sections, Table 1 summarizes the main AI approaches applied to nanopharmaceutical development, while Figure 3 presents a comparative synthesis of key modeling strategies and their translational relevance.

To increase the practical value of this review and facilitate implementation by readers, representative models, tools, and computational platforms associated with each AI-based approach are also included in Table 1. These examples include commonly used machine learning libraries, deep learning frameworks, graph-based modeling tools, optimization environments, explainable AI methods, and mechanistic or hybrid modeling platforms. Although the listed tools are not exhaustive, they illustrate how different AI strategies can be selected according to the type of available data, the intended prediction task, and the stage of nanopharmaceutical development.

To complement this methodological overview, Table 2 summarizes selected examples from the literature demonstrating how AI-based approaches have been practically applied in nanopharmaceutical research. These examples include formulation–property prediction, active learning-guided nanoparticle optimization, protein corona and biological fate prediction, pharmacokinetic modeling, and toxicity assessment. Importantly, the table also highlights the level of validation performed in each case, ranging from retrospective validation using literature-derived datasets to experimental feedback loops and comparison with measured pharmacokinetic or biological data.

Rather than being interchangeable, AI approaches differ substantially in their data requirements, interpretability, maturity, and suitability for specific nanopharmaceutical tasks. Supervised machine learning is currently most suitable for structured formulation datasets and prediction of critical quality attributes such as particle size, polydispersity, zeta potential, encapsulation efficiency, drug loading, release behavior, and stability. Deep learning is more appropriate for large, complex, or unstructured datasets, including microscopy images, omics data, temporal release profiles, and multimodal biological assays, but it requires larger annotated datasets and stronger interpretability strategies. Bayesian optimization and evolutionary algorithms are most useful when the goal is adaptive or multi-objective formulation optimization, particularly when experimental resources are limited or multiple performance criteria must be balanced. Physics-informed and hybrid mechanistic–AI models are especially valuable when mechanistic knowledge of release, transport, pharmacokinetics, or manufacturing processes is available, because they can improve extrapolation and regulatory confidence. Reinforcement learning, patient-specific digital twins, and fully autonomous closed-loop systems remain promising but less mature for nanopharmaceutical applications and require stronger experimental, manufacturing, and clinical validation before broad implementation.

Although the representative studies summarized in Table 2 demonstrate the practical value of AI-based approaches, reported performance metrics are not yet standardized across nanopharmaceutical applications. Some formulation studies provide mainly retrospective prediction outputs or graphical validation rather than uniform metrics such as R^2^, RMSE, sensitivity, or specificity [26]. In contrast, studies combining design of experiments with machine learning for PLGA nanoparticle optimization have reported improved predictive performance for particle size using machine learning models, as reflected by lower RMSE values and higher coefficients of determination compared with classical design-of-experiments models, although zeta potential remained more difficult to predict accurately [30]. More quantitative validation has been reported for AI-assisted pharmacokinetic modeling; for example, an AI-assisted PBPK model predicted nanoparticle tumor delivery with R^2^ = 0.83 and RMSE = 3.01 for DE24, R^2^ = 0.56 and RMSE = 2.27 for DE168, and R^2^ = 0.82 and RMSE = 3.51 for DEmax, while also showing R^2^ ≥ 0.70 for 133 of 288 experimentally measured pharmacokinetic datasets [18]. These examples indicate that AI performance should be interpreted in relation to the endpoint, dataset size, validation design, and biological complexity of the prediction task. Future studies should therefore report standardized performance metrics, uncertainty estimates, external validation results, and applicability domains to allow meaningful comparison across AI models and nanopharmaceutical platforms.

### 2.1. Machine Learning for Formulation–Property Relationships

Machine learning has become a central strategy for modeling quantitative relationships between formulation variables and nanopharmaceutical properties [17,23,29]. Unlike conventional empirical approaches, which often evaluate factors individually or within narrow experimental domains, machine learning can analyze multiple interdependent variables simultaneously and identify nonlinear patterns that influence critical quality attributes. This is particularly relevant for nanopharmaceutical systems, where composition, preparation method, process parameters, and material attributes collectively determine product performance [20,30].

Supervised learning algorithms, including random forests, support vector machines, gradient boosting methods, artificial neural networks, and ensemble models, have been used to predict particle size, polydispersity index, zeta potential, encapsulation efficiency, drug loading, colloidal stability, and release behavior [17,23,29]. In polymeric nanoparticle development, PLGA-based datasets have been used to train machine learning models for predicting formulation attributes such as particle size, PDI, drug loading, encapsulation efficiency, and zeta potential, thereby supporting early formulation screening and optimization [17,29,30]. In contrast, lipid nanoparticle studies have focused more strongly on formulation and process development, including the identification of design features associated with transfection efficiency and cell-type-preferential delivery [31,36]. These examples show that AI applications differ according to nanocarrier class, dataset structure, and intended performance endpoint.

However, accurate prediction of physicochemical critical quality attributes should not be interpreted as direct evidence of biological or clinical performance. Parameters such as particle size, polydispersity index, zeta potential, encapsulation efficiency, and drug loading are essential for formulation screening, but they are only indirect predictors of in vivo behavior. For example, even when nanoparticle systems are rationally designed and physicochemically optimized, quantitative analyses of nanoparticle delivery to solid tumors have shown that only a small fraction of the injected dose reaches the target site, with reported median tumor delivery efficiencies of approximately 0.7% of the injected dose [39]. This illustrates a major translational gap between CQA optimization and actual biological performance. Therefore, AI models should increasingly integrate biological endpoints from the start, including protein corona formation, cellular uptake, biodistribution, pharmacokinetics, toxicity, immunogenicity, and therapeutic response, rather than focusing only on formulation-level descriptors [4,21,22,38,39,40,41,42,43].

Different algorithms offer distinct advantages depending on dataset size, complexity, and modeling objective. Tree-based and ensemble models are useful for capturing interaction effects and ranking formulation variables, support vector machines may perform well in smaller datasets, and neural networks may be more suitable for larger and more complex datasets [20,32,33]. Comparative studies combining design of experiments and machine learning suggest that ML models, particularly gradient boosting approaches, may outperform classical design of experiments (DoE) in predicting nanoparticle size and identifying influential formulation parameters; however, DoE remains valuable for generating structured experimental datasets and defining the initial design space [33]. Thus, ML should be viewed as complementary to, rather than a replacement for, statistically designed experimentation.

Machine learning can also extend the value of design of experiments by generating predictive maps of the formulation space and supporting multi-attribute optimization, where particle size, drug loading, encapsulation efficiency, stability, release kinetics, and biological compatibility must be balanced simultaneously [20,33,34]. This is particularly important in lipid nanoparticle and polymeric nanoparticle development, where formulation performance depends on coupled effects among material composition, processing conditions, and biological endpoints [31,36].

Despite these advantages, machine learning models should not be interpreted as universally reliable predictors. Many studies rely on limited datasets, inconsistent experimental conditions, or incomplete descriptor reporting, which restricts generalizability [20,34]. Good internal validation does not guarantee robustness across laboratories, nanocarrier platforms, preparation methods, or biological environments. Therefore, external validation, sensitivity analysis, uncertainty estimation, and transparent reporting are essential.

Feature selection and interpretability are also important for translating machine learning predictions into formulation knowledge. Tools such as SHAP, permutation importance, partial dependence analysis, and feature ablation can identify variables that most strongly influence model outputs, such as particle size, polymer concentration, lipid composition, surfactant type, surface charge, or processing energy [20,27,33]. However, statistical importance should not be interpreted as direct evidence of causality without experimental confirmation. For translational use, model-derived design rules must be experimentally tested and evaluated under relevant formulation, manufacturing, and biological conditions.

Overall, machine learning provides a valuable framework for improving formulation–property modeling in nanopharmaceutical development. Its main contribution is not only accelerating formulation screening, but also transforming fragmented experimental observations into predictive and interpretable design rules that can support formulation optimization, quality-by-design strategies, manufacturing scale-up, and regulatory decision-making [20,33,34].

Representative implementation environments for these models include open-source machine learning libraries such as scikit-learn, XGBoost, and LightGBM, as well as MATLAB-based toolboxes, which can support model training, cross-validation, hyperparameter optimization, feature ranking, and formulation–property prediction. For example, literature-derived PLGA nanoparticle datasets have been used to train machine learning models capable of predicting particle size, PDI, drug loading, and encapsulation efficiency, demonstrating how AI can support early formulation screening and reduce empirical trial-and-error development [26].

### 2.2. Deep Learning for Complex Data Integration

Deep learning extends conventional machine learning by enabling the automatic extraction of relevant features from complex, high-dimensional, and unstructured datasets [21,22,35]. This is particularly useful in nanopharmaceutical research, where important information is often embedded in microscopy images, spectroscopic profiles, high-throughput screening outputs, release curves, omics data, and multiparametric biological assays [35,36,37]. By learning directly from raw or minimally processed data, deep learning can identify patterns that may not be captured by manually selected descriptors or conventional statistical methods.

In nanopharmaceutical development, deep learning is especially valuable for image-based characterization and temporal modeling. Convolutional neural networks and object-detection architectures have been applied to microscopy-based nanoparticle analysis, including particle detection, size measurement, morphology classification, segmentation, and the automated evaluation of nanostructures [35,36].

Compared with manual or threshold-based image analysis, these approaches can improve throughput and reduce operator bias, but they remain sensitive to image quality, annotation strategy, microscope settings, and dataset diversity [35,36]. Other architectures, including recurrent neural networks, long short-term memory networks, temporal convolutional networks, and transformers, can model time-dependent processes such as drug release, nanoparticle degradation, cellular uptake, pharmacokinetics, and longitudinal toxicity responses [38,39].

Another important advantage of deep learning is its capacity for multimodal data integration. Nanopharmaceutical datasets increasingly combine physicochemical descriptors, imaging outputs, spectroscopic signatures, molecular structures, biological assays, and pharmacokinetic measurements [21,22,37]. Deep learning models can integrate these heterogeneous data sources into unified predictive frameworks, helping to connect nanoscale design features with biological outcomes. This is particularly relevant for nano–bio interactions, which depend on the combined influence of nanoparticle properties, protein adsorption, cellular phenotype, immune response, and tissue microenvironment.

Despite its potential, deep learning remains limited by the scarcity of large, diverse, and well-annotated datasets in nanopharmaceutical research [21,35,36]. Many models show strong internal performance but fail to generalize across laboratories, imaging conditions, biological models, or nanocarrier platforms. Interpretability is also a major challenge, since black-box predictions may reflect dataset-specific artifacts rather than biologically meaningful features. Therefore, the translational use of deep learning requires standardized datasets, transparent reporting, explainability tools, uncertainty estimation, external validation, and experimental confirmation [35,36,40].

In practical applications, deep learning workflows for nanopharmaceutical and related materials datasets can support image-based characterization, microscopy analysis, multimodal data integration, temporal modeling, and scalable model development. These workflows are particularly relevant for nanoparticle image analysis, electron microscopy interpretation, high-content biological assays, and time-dependent pharmacokinetic or release-related datasets [35,36,38,39].

### 2.3. Physics-Informed and Hybrid Modeling Approaches

Although data-driven models have improved prediction in nanopharmaceutical research, their limitations become evident when models are applied beyond the experimental conditions represented in the training dataset [41,42]. Machine learning and deep learning can identify correlations between formulation variables and performance outcomes, but they often lack mechanistic consistency, interpretability, and extrapolation capacity. This limitation is particularly relevant in nanopharmaceutical development, where changes in scale, raw materials, processing conditions, biological media, or administration route can substantially alter system behavior.

Physics-informed and hybrid modeling approaches address these limitations by integrating mechanistic principles into data-driven frameworks. Instead of relying exclusively on empirical correlations, these models incorporate knowledge of diffusion, dissolution, degradation, mass transfer, colloidal stability, transport phenomena, pharmacokinetics, and biological barriers [41,42,43]. Compared with purely data-driven models, hybrid approaches can constrain predictions within scientifically meaningful boundaries, reduce unrealistic outputs, and improve model robustness under variable experimental and manufacturing conditions.

Hybrid models are especially useful for drug release, pharmacokinetic, biodistribution, and manufacturing applications. In drug release studies, they can combine classical release equations with machine learning to account for formulation-specific variability, complex matrices, and drug–carrier interactions. In pharmacokinetic and biodistribution modeling, machine learning can complement physiologically based pharmacokinetic models by capturing nanoparticle-specific behaviors such as protein corona formation, mononuclear phagocyte system uptake, organ accumulation, and size- or surface-dependent clearance [44,45]. For example, AI-assisted PBPK models have been proposed to predict nanoparticle delivery efficiency from physicochemical properties, illustrating how mechanistic structure and data-driven correction can be combined for more translationally relevant prediction [44].

In manufacturing, physics-informed and hybrid models can support the prediction of critical quality attributes by incorporating process-dependent phenomena such as mixing, nucleation, aggregation, solvent exchange, shear stress, and temperature effects [41,46]. Compared with conventional empirical process models, these approaches are more compatible with digital twins and process analytical technology because they can link real-time data streams with mechanistic process understanding [46,47].

Despite their advantages, physics-informed and hybrid models are more complex to develop than standard machine learning pipelines. They require reliable mechanistic knowledge, robust parameter estimation, interdisciplinary expertise, uncertainty quantification, and external validation [41,42,46]. However, from a translational perspective, these models are particularly valuable because they provide a clearer rationale linking formulation composition, manufacturing parameters, critical quality attributes, and biological performance. Therefore, they are better positioned to support quality-by-design strategies, process analytical technology, digital twins, regulatory evaluation, manufacturing scale-up, and clinical translation.

Representative physics-informed and hybrid modeling strategies include physics-informed neural networks, differential equation-constrained learning, mechanistic–machine learning process models, machine learning-assisted PBPK frameworks, and hybrid models designed to support digital twin development. These approaches are useful because they integrate data-driven prediction with mechanistic knowledge of diffusion, transport phenomena, pharmacokinetics, and pharmaceutical process behavior, thereby improving interpretability and extrapolation compared with purely empirical models [28,29,30,31,41,42,43,44,45,46].

### 2.4. Digital Twins for Nanopharmaceutical Development

Digital twins are dynamic virtual representations of physical systems that are continuously updated with experimental, manufacturing, or real-time process data [48,49]. In nanopharmaceutical development, they may simulate formulation behavior, predict product performance, monitor manufacturing variability, and guide optimization of critical process conditions. Unlike static computational models, digital twins evolve as new data become available, making them particularly relevant for systems whose properties are highly sensitive to raw materials, formulation composition, mixing, temperature, solvent exchange, shear stress, equipment configuration, and scale-up parameters.

By integrating formulation variables, process analytical technology data, machine learning models, mechanistic simulations, and quality-by-design principles, digital twins can connect formulation design, process monitoring, and product performance within a single predictive framework [48,49,50]. Compared with process analytical technology (PAT) used only as a monitoring tool, digital twins may provide a more integrated structure for real-time prediction, scenario testing, and decision support. This allows developers to estimate how changes in process conditions affect critical quality attributes such as particle size, polydispersity, zeta potential, encapsulation efficiency, drug loading, release kinetics, and colloidal stability. In this way, digital twins may shift nanopharmaceutical manufacturing from retrospective quality assessment toward real-time prediction, prevention, and control.

This capability is especially relevant during scale-up. Formulations optimized at laboratory scale often fail to maintain the same physicochemical and biological properties during pilot or industrial production because of changes in mixing efficiency, batch volume, energy input, flow dynamics, sterilization conditions, and equipment geometry. Digital twins may reduce this uncertainty by simulating scale-dependent effects, supporting technology transfer, and identifying process conditions that preserve formulation quality across manufacturing scales [49,50,51]. They can also support scenario testing and predictive troubleshooting, reducing experimental burden, material consumption, development time, and the risk of process failure.

Despite their potential, digital twins remain at an early stage in nanopharmaceutical development. Their implementation requires high-quality input data, mechanistic understanding, validated predictive models, real-time data acquisition systems, and continuous feedback between physical and virtual environments [48,49,52]. Fragmented datasets, poorly standardized process descriptors, and limited comparability across laboratories may restrict model accuracy and transferability. Regulatory use also requires external validation, uncertainty quantification, lifecycle management, and clear documentation of model assumptions, update procedures, and performance boundaries [47,51].

Overall, digital twins offer a promising path toward more robust and scalable nanopharmaceutical development by linking formulation design, manufacturing control, quality assurance, and predictive performance assessment. When combined with AI, process analytical technology, mechanistic modeling, and quality-by-design frameworks, they may help bridge the gap between optimized laboratory formulations and reproducible, scalable, and clinically translatable nanopharmaceutical products [47,48,49,50,51,52].

In practical implementation, digital twin frameworks in pharmaceutical and biopharmaceutical manufacturing commonly integrate mechanistic process models, process analytical technology data, and data-driven modules for real-time monitoring, scenario testing, scale-up support, and process decision-making. Although specific applications in nanopharmaceutical manufacturing remain less mature, this integration provides a translational basis for predicting how process parameters influence critical quality attributes and for supporting continuous manufacturing, lifecycle control, and regulatory-aligned development [47,48,49,50,51,52].

### 2.5. Data-Driven Optimization of Nanopharmaceutical Formulations

The optimization of nanopharmaceutical formulations is inherently complex because product performance depends on interdependent formulation variables, material attributes, processing conditions, and biological responses [20,32,33]. Parameters such as polymer or lipid composition, drug-to-carrier ratio, surfactant concentration, solvent system, mixing rate, temperature, particle size, surface charge, encapsulation efficiency, and release kinetics often influence one another in nonlinear ways. As a result, nanopharmaceutical optimization should be viewed not as the adjustment of isolated parameters, but as a multi-attribute decision-making problem involving stability, manufacturability, biodistribution, toxicity, and therapeutic efficacy [20,53].

Traditional one-factor-at-a-time approaches are poorly suited to this complexity because they do not capture interactions between formulation and process variables and may lead to local rather than global optima. Design of experiments and response surface methodology provide a more structured exploration of formulation space, allowing the identification of main effects, interaction effects, and optimized experimental regions [23,24]. However, these approaches may still be limited when relationships are highly nonlinear or when physicochemical, biological, pharmacokinetic, and manufacturing data must be integrated. Compared with classical design of experiments (DoE)/response surface methodology (RSM), AI-enabled optimization strategies offer greater flexibility for learning formulation–process–performance relationships from complex datasets, although they remain dependent on data quality, experimental coverage, and validation beyond the initial design space [20,33,53].

Data-driven optimization can generate predictive maps of the design space, identify promising formulation regions, avoid unstable or unsafe compositions, and prioritize experiments with high informational value. Machine learning, Bayesian optimization, genetic algorithms, ensemble methods, and reinforcement learning can support this process by accommodating nonlinear interactions, high-dimensional variables, and multiple performance endpoints [20,53,54,55]. Bayesian optimization is particularly useful when experiments are costly because it balances exploration of uncertain regions with exploitation of promising candidates, whereas evolutionary algorithms are useful for broad multi-objective searches but may generate solutions that require additional manufacturability constraints [53,54]. Reinforcement learning may support sequential formulation or process decisions, but its application in nanopharmaceutical development remains less mature and requires stronger experimental validation [55].

The integration of AI with design of experiments and quality-by-design principles enables an iterative optimization workflow in which experimental data are generated, modeled, interpreted, and used to guide subsequent formulation or process selection [10,23,24,33]. Each cycle can refine the design space, reduce unnecessary experiments, and improve the probability of identifying robust and reproducible formulations. From a translational perspective, this approach can also support risk assessment by identifying formulation and process variables that most strongly influence critical quality attributes. However, AI-guided optimization should not be evaluated only by mathematical performance; optimized formulations must also demonstrate biological relevance, process robustness, scalability, and regulatory compatibility [10,47,51]. To summarize these strategies, Table 3 presents the main data-driven approaches supporting nanopharmaceutical optimization, quality control, and manufacturing translation.

However, data-driven optimization must be interpreted critically. Its reliability depends directly on dataset quality, experimental consistency, descriptor standardization, and model validation [20,25,53]. Sparse, biased, or poorly annotated datasets may lead to misleading correlations, poorly reproducible recommendations, or overfitting to narrow experimental conditions. This limitation is particularly important for Bayesian optimization, evolutionary algorithms, and reinforcement learning, which may efficiently explore a design space but still depend on how well that space was experimentally defined and validated [53,54,55]. Therefore, AI-guided optimization should include uncertainty estimation, external validation, experimental confirmation, and transparent definition of model boundaries.

Mathematical optimality also does not guarantee biological or clinical relevance. A formulation optimized for physicochemical attributes may still fail because of unfavorable protein corona formation, rapid clearance, immune activation, toxicity, poor target accumulation, or limited manufacturability [4,18,19]. Thus, advanced optimization workflows should progressively incorporate biological, pharmacokinetic, toxicological, process-related, and translational endpoints rather than focusing only on particle size, encapsulation efficiency, or release kinetics.

Overall, data-driven optimization can support more rational nanopharmaceutical development by enabling the systematic exploration of complex design spaces, identification of variable interactions, prediction of critical quality attributes, and balancing of competing objectives [20,53,54,55]. Future studies should move beyond isolated optimization examples and demonstrate externally validated workflows that connect formulation design with manufacturing scalability, quality control, regulatory requirements, and clinical performance [47,51].

A practical example of this strategy is the use of machine learning-guided high-throughput nanoparticle design, in which experimental nanoparticle preparation, microfluidic formulation, and high-content imaging were combined with iterative model-guided optimization. This type of workflow demonstrates how AI can move beyond retrospective prediction and actively guide experimental formulation development, supporting improved nanoparticle performance through feedback between computational models and experimental validation [37].

### 2.6. Integration of AI with Design of Experiments

Design of experiments remains a key methodological foundation for pharmaceutical formulation development because it enables the structured evaluation of multiple formulation and process variables within a defined experimental space [23,24]. Compared with one-factor-at-a-time strategies, DoE provides a more efficient and statistically rigorous approach for identifying main effects, interaction effects, and response surfaces associated with critical quality attributes [10,23]. This is particularly relevant for nanopharmaceuticals, where variables such as carrier composition, drug concentration, surfactant level, solvent ratio, mixing speed, temperature, and processing time often interact in nonlinear ways [23,30].

However, classical DoE may be limited when applied to complex nanopharmaceutical systems. Factorial, fractional factorial, central composite, and Box–Behnken designs are useful for exploring predefined formulation spaces, but they may be less effective when many variables, nonlinear relationships, biological endpoints, pharmacokinetic behavior, toxicity, and manufacturing constraints must be considered simultaneously [23,24]. In these cases, DoE is often more suitable for local optimization than for broader predictive modeling. Therefore, its value increases when it is combined with AI models capable of extending prediction across more complex formulation–process–performance relationships [20,30].

The integration of AI with DoE can overcome some of these limitations. DoE provides structured and high-quality experimental datasets, while AI models can extract nonlinear patterns, predict critical quality attributes, identify optimal regions, and detect variable interactions that may not be fully captured by classical statistical models [20,30]. Comparative work combining DoE and machine learning in polymeric nanoparticle optimization suggests that ML can improve prediction of formulation attributes, whereas DoE remains essential for defining a rational and experimentally controlled design space [33]. Thus, AI should be viewed as complementary to DoE rather than a replacement for it.

In adaptive workflows, initial experiments are used to train a preliminary model, and subsequent experiments are selected based on model predictions, uncertainty estimates, or expected improvement [53]. This creates a closed-loop optimization process in which each experimental cycle informs the next. AI-assisted DoE can reduce experimental burden by prioritizing the most informative experiments and progressively refining the design space. This is especially valuable for nanopharmaceuticals, where each experiment may require extensive physicochemical characterization, stability testing, biological evaluation, and material consumption.

However, the approach must be implemented carefully. Narrow, biased, or poorly distributed initial designs may lead to misleading predictions, particularly outside the sampled experimental domain [20,33,53]. Therefore, uncertainty quantification, independent validation, and experimental confirmation of predicted optima are essential. Another important consideration is the selection of meaningful response variables. Many AI–DoE studies focus on physicochemical endpoints such as particle size, polydispersity index, zeta potential, and encapsulation efficiency. Although these parameters are essential, they are not sufficient to determine translational potential. Future AI-assisted DoE frameworks should also incorporate biological compatibility, release behavior, pharmacokinetics, toxicity, stability, and manufacturability endpoints.

Overall, integrating AI with DoE can transform formulation optimization from a static experimental exercise into an iterative and data-driven workflow. DoE contributes experimental structure and statistical rigor, whereas AI adds predictive flexibility, nonlinear modeling capacity, and adaptive decision-making [20,23,33,53]. For translational relevance, AI-assisted DoE should generate formulations that are not only statistically optimized, but also reproducible, scalable, biologically relevant, and clinically meaningful.

In practice, the combination of DoE and machine learning has been applied to nanoparticle formulation development by using structured experimental designs to generate high-quality training data and predictive algorithms to model formulation attributes. For example, studies combining DoE with machine learning in PLGA nanoparticle production have shown that this integrated strategy can improve the prediction of particle size and formulation performance while preserving the statistical structure and experimental control provided by DoE [23,24,33,53].

### 2.7. Multi-Objective Optimization

Nanopharmaceutical development is inherently a multi-objective optimization problem because an effective formulation must satisfy performance, safety, and manufacturing criteria simultaneously [20,53,54]. In most cases, improving one attribute may compromise another. For example, increasing drug loading can reduce colloidal stability or accelerate burst release, whereas reducing particle size may improve tissue penetration or cellular uptake but alter biodistribution, clearance, toxicity, or manufacturing reproducibility. Therefore, formulation development requires balancing particle size, polydispersity, zeta potential, drug loading, encapsulation efficiency, release kinetics, stability, toxicity, pharmacokinetics, scalability, and batch-to-batch reproducibility.

Multi-objective optimization algorithms provide a useful framework for managing these trade-offs. Approaches such as Bayesian optimization, genetic algorithms, particle swarm optimization, evolutionary strategies, and reinforcement learning can explore complex formulation spaces more efficiently than exhaustive experimental screening [53,54,55]. Bayesian optimization is particularly useful in data-limited settings because it combines prediction with uncertainty estimation, while evolutionary algorithms and particle swarm optimization are useful for broader searches across competing objectives [53,54]. Reinforcement learning may support sequential decision-making in closed-loop systems, although its use in nanopharmaceutical development remains less mature and requires stronger experimental validation [55].

A central concept in this context is the identification of Pareto-optimal solutions, in which one objective cannot be improved without worsening another [53,54]. This is highly relevant for nanopharmaceuticals because the most suitable formulation is not necessarily the one that maximizes a single parameter, but the one that provides the most acceptable trade-off for a specific therapeutic application, route of administration, manufacturing process, and regulatory context. Pareto analysis can help visualize these trade-offs and support more transparent formulation selection.

AI-enabled multi-objective optimization is particularly valuable because it can integrate heterogeneous endpoints, including physicochemical attributes, process parameters, biological responses, toxicity indicators, and pharmacokinetic outcomes [20,53]. However, optimization quality depends strongly on how objectives are defined, weighted, constrained, and validated. A formulation optimized only for particle size, zeta potential, or encapsulation efficiency may still fail because of unfavorable protein corona formation, immune activation, poor pharmacokinetics, toxicity, or limited manufacturability [4,18,19]. Therefore, optimization frameworks should include biological endpoints, process feasibility, scalability, robustness, regulatory acceptability, and batch reproducibility [47,51].

Overall, multi-objective optimization is a critical component of AI-driven nanopharmaceutical development because it enables transparent analysis of trade-offs among formulation, biological, and manufacturing objectives. Future studies should move beyond reporting mathematically optimized formulations and demonstrate that selected candidates retain their performance under independent validation, biologically relevant testing, and scalable manufacturing conditions.

These practical examples highlight that AI-guided optimization should be evaluated not only by mathematical prediction accuracy, but also by experimentally validated improvements in formulation performance, biological response, and manufacturability. Therefore, future multi-objective optimization workflows should combine computational prediction with experimental confirmation, ensuring that selected formulations represent feasible and translationally relevant solutions rather than only mathematically optimal candidates [37,53,54,55].

### 2.8. Process Analytical Technology and Real-Time Control

Process analytical technology plays a central role in the transition from laboratory-scale nanopharmaceutical development to reproducible and scalable manufacturing [12]. Because nanopharmaceutical products are highly sensitive to variations in mixing rate, temperature, flow conditions, pressure, solvent exchange, homogenization energy, and raw material properties, real-time monitoring is essential for maintaining critical quality attributes such as particle size, polydispersity, zeta potential, drug loading, encapsulation efficiency, aggregation tendency, and release behavior [10,12].

Traditional quality control relies largely on end-product testing, which detects failures only after manufacturing is complete. For complex nanopharmaceutical systems, this can lead to batch rejection, material loss, delayed development, and limited process understanding. In contrast, process analytical technology enables real-time or near-real-time monitoring of critical process parameters and quality attributes, allowing deviations to be identified and corrected during manufacturing [12,51].

The integration of artificial intelligence with process analytical technology expands this capability from passive monitoring to predictive control [47,56]. Machine learning models can analyze sensor outputs, spectroscopic data, particle characterization signals, flow measurements, temperature profiles, and process metadata to identify patterns associated with product quality. Compared with conventional monitoring systems, AI-assisted PAT can detect anomalies, predict deviations, and recommend corrective actions before product attributes move outside the desired design space [47,56].

This approach is particularly relevant for nanopharmaceutical manufacturing because nanoparticle formation often involves rapid and dynamic processes such as nucleation, self-assembly, precipitation, emulsification, solvent diffusion, aggregation, and stabilization. AI-assisted process analytical technology can help link these process events to final product attributes, improving understanding of how manufacturing conditions influence nanocarrier quality and supporting scale-up [12,47,51].

Real-time control also strengthens quality-by-design implementation. By modeling nonlinear relationships among critical material attributes, critical process parameters, and critical quality attributes, AI can support dynamic process adjustment and reduce batch-to-batch variability [10,12]. This is especially important during technology transfer, where formulations optimized at small scale may not behave identically in pilot or industrial production. When integrated with digital twins, PAT data can also support scenario testing, predictive troubleshooting, and continuous process refinement [48,49,50,51,52].

Despite its potential, AI-assisted process analytical technology requires robust sensors, reliable data streams, validated analytical methods, and predictive models that remain accurate under manufacturing conditions. Models developed from small laboratory datasets may not generalize to larger-scale production, where process variability is greater. Therefore, external validation, uncertainty estimation, lifecycle monitoring, and continuous model verification are essential [47,51,56].

Regulatory implementation also requires transparency. For AI-based process control to be accepted in regulated environments, developers must document how models are trained, validated, updated, monitored, and used to support manufacturing decisions [47]. Overall, the integration of AI with process analytical technology can support real-time quality control, reduce batch variability, improve scale-up, and strengthen the manufacturing robustness of nanopharmaceutical products.

In practical manufacturing contexts, AI-assisted process analytical technology can combine real-time spectroscopic, sensor-based, and process metadata with predictive models to detect deviations, estimate product quality, and support adaptive process control. Although direct nanopharmaceutical case studies remain limited, pharmaceutical PAT applications using chemometrics and machine learning provide an important translational foundation for monitoring critical process parameters and critical quality attributes, particularly when integrated with QbD, digital twins, and continuous manufacturing frameworks [12,47,51,56].

### 2.9. Quality-by-Design and Regulatory Alignment

Quality-by-design is a central framework for improving the development, manufacturing, and regulatory evaluation of nanopharmaceutical products [10,11]. Its main principle is that product quality should be built into the formulation and manufacturing process from the beginning, rather than confirmed only through final product testing [10,57]. This is particularly important for nanopharmaceuticals, where small variations in material attributes or process conditions can affect critical quality attributes such as particle size, polydispersity, surface charge, encapsulation efficiency, drug loading, release kinetics, sterility, stability, and biological performance.

Within a quality-by-design framework, the identification of critical material attributes, critical process parameters, and critical quality attributes is essential for defining a reliable design space [10,57]. However, this is challenging in nanopharmaceutical development because product performance depends on nonlinear interactions among formulation composition, preparation method, process conditions, physicochemical properties, and biological responses [20,30]. Classical QbD tools provide structure, but they may not fully capture this complexity, especially when biological endpoints, pharmacokinetics, toxicity, and manufacturing scalability are considered.

Artificial intelligence can strengthen QbD by transforming experimental and process data into predictive knowledge. Machine learning models can identify variables that most strongly influence product quality, predict critical quality attributes under different conditions, and support the definition of more robust design spaces [20,33,56]. In this sense, AI does not replace QbD, but expands its predictive capacity by modeling nonlinear and high-dimensional relationships that are difficult to capture using conventional tools.

The integration of AI and QbD is also relevant for risk assessment and regulatory alignment. Predictive models can help identify high-risk formulation regions, process conditions associated with instability, and variables that contribute to batch variability [47,57]. This can support proactive quality management, justify control strategies, and provide quantitative evidence linking formulation variables, process parameters, and product performance. For nanopharmaceuticals, this is particularly valuable because regulatory evaluation often requires detailed evidence of reproducibility, physicochemical stability, manufacturing consistency, and biological safety.

However, regulatory use of AI-assisted QbD requires more than predictive accuracy. Models must be interpretable, externally validated, clearly documented, and supported by uncertainty analysis [47,58]. Regulatory agencies need to understand how predictions are generated, which variables drive model outputs, what assumptions are embedded in the model, and under which conditions the model remains reliable. Model lifecycle management is also essential, including procedures for monitoring, revalidation, version control, performance tracking, and documentation of updates [47,58].

Overall, the integration of AI with QbD offers a promising pathway for improving the robustness, scalability, and regulatory readiness of nanopharmaceutical products. Its main contribution is the connection of formulation design, process understanding, risk assessment, and quality control within a predictive framework [10,47,57,58]. Future studies should demonstrate validated AI-QbD workflows that support reproducible manufacturing, robust design-space definition, transparent decision-making, and clinically relevant regulatory evidence.

From an implementation perspective, AI-assisted QbD workflows can support the identification of critical material attributes, critical process parameters, and critical quality attributes by combining experimental design data, process monitoring outputs, and predictive modeling. However, for regulatory relevance, these models should be accompanied by clear documentation of the intended use, input variables, training datasets, validation strategy, uncertainty analysis, applicability domain, and lifecycle management procedures. This is particularly important when AI outputs are used to support process control, risk assessment, design-space justification, or regulatory decision-making [10,12,16,47,57,58].

Overall, AI-based modeling approaches can support nanopharmaceutical design by connecting formulation variables, material attributes, process parameters, and critical quality attributes within predictive frameworks. Machine learning, deep learning, physics-informed models, digital twins, design of experiments, quality-by-design, process analytical technology, and multi-objective optimization each provide complementary strengths, ranging from formulation–property prediction to process monitoring and regulatory-aligned development. However, their practical value depends on dataset quality, external validation, interpretability, uncertainty analysis, and experimental confirmation.

## 3. AI in Nano–Bio Interactions, Pharmacokinetics, and Precision Nanomedicine

### 3.1. AI in Nano–Bio Interactions and Pharmacokinetics

The biological performance of nanopharmaceutical systems is determined by dynamic and context-dependent interactions at the nano–bio interface [4,18,19,59]. After administration, nanocarriers interact with biological fluids, proteins, lipids, immune components, cellular barriers, extracellular matrices, and organ-specific microenvironments. These interactions can modify the original physicochemical identity of the nanoparticle and influence protein corona formation, cellular uptake, intracellular trafficking, immune recognition, biodistribution, pharmacokinetics, toxicity, and therapeutic efficacy.

A central challenge is that biological performance cannot be predicted from physicochemical properties alone. Although size, surface charge, hydrophobicity, morphology, stiffness, composition, and surface functionalization are important, their effects depend strongly on the biological context, including administration route, protein composition of the medium, cell type, disease state, tissue architecture, immune status, and species-specific physiology [4,7,18,59]. This context dependence helps explain why many nanopharmaceuticals with promising in vitro performance fail to reproduce their effects in animal models or clinical settings [7,9].

This limitation is particularly important for AI-driven formulation design, because models trained only to predict physicochemical CQAs may identify formulations that appear optimal in vitro but fail to achieve meaningful in vivo performance. Future models should therefore link formulation descriptors and CQAs with biological endpoints such as protein corona composition, cellular uptake, biodistribution, pharmacokinetic exposure, immune activation, toxicity, and therapeutic efficacy. This integrated strategy would help shift AI from formulation-level optimization toward biologically informed and translationally relevant nanopharmaceutical prediction [4,21,22,38,39,40,41,42,43].

AI can support this field by integrating physicochemical descriptors with imaging, omics, toxicological, pharmacokinetic, and clinical data [13,18,19,40,60]. Machine learning and deep learning models may help predict protein corona patterns, cellular uptake, intracellular trafficking, organ accumulation, clearance, toxicity, immunogenicity, and patient-specific responses. Compared with conventional approaches that often evaluate these processes separately, AI offers the possibility of linking nanoscale design features with biological outcomes across multiple levels of complexity. However, most current studies remain focused on specific endpoints, such as corona composition, uptake, or toxicity, whereas integrated models that jointly capture nano–bio interactions, pharmacokinetics, and translational performance are still limited [18,19,60].

**Figure 4:**
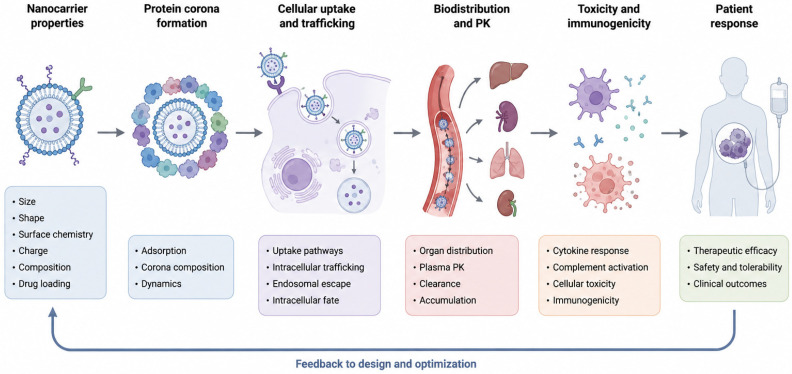
AI-enabled modeling of nano–bio interactions and biological outcomes. The figure illustrates how nanocarrier properties influence protein corona formation, cellular uptake and intracellular trafficking, biodistribution and pharmacokinetics, toxicity and immunogenicity, and patient response. This workflow highlights the need to connect physicochemical descriptors with biological endpoints to improve translational prediction and guide formulation optimization.

By connecting nanoscale design features with biological outcomes, AI provides a framework for improving safer-by-design formulation, translational prediction, and precision nanomedicine. To summarize these applications, Table 4 presents the main biological domains in which AI can support nano–bio interaction modeling, pharmacokinetic prediction, safety assessment, and clinical translation. Figure 4 further illustrates how AI can connect nanoparticle design features with protein corona formation, cellular uptake, intracellular trafficking, biodistribution, pharmacokinetics, toxicity, immunogenicity, and precision nanomedicine.

AI-driven nano–bio modeling is important because protein corona formation, cellular uptake, biodistribution, pharmacokinetics, toxicity, and immunogenicity are mechanistically interconnected [4,18,19,59,60,61]. Changes in nanoparticle size, surface charge, composition, or surface chemistry may alter biological identity, cellular recognition, immune clearance, tissue accumulation, and therapeutic response [60,61,62]. Therefore, conventional assays that evaluate these processes separately may provide only a partial view of nanopharmaceutical behavior.

By integrating physicochemical descriptors with omics, imaging, toxicological, pharmacokinetic, and biological datasets, AI models can identify nonlinear relationships between nanoparticle properties and biological outcomes [13,20,35,62,64]. As illustrated in Figure 4, these approaches can connect nanoscale formulation attributes with responses across molecular, cellular, tissue, and organism levels.

However, AI-based nano–bio modeling requires careful validation. Biological datasets are often influenced by differences in cell lines, animal models, protein sources, assay protocols, imaging methods, and dose metrics [18,19,64,65]. If these variables are not properly reported, models may learn dataset-specific artifacts rather than biologically meaningful relationships. Future studies should prioritize standardized biological datasets, mechanistically informed modeling, uncertainty quantification, external validation, and clinically relevant pharmacokinetic endpoints [62,64,65].

For instance, supervised learning combined with mass spectrometry-based protein corona analysis has been used to predict the in vivo fate of nanomaterials, demonstrating that the biological identity formed at the nanoparticle surface can provide predictive information on biodistribution and biological behavior. This example illustrates how AI can move beyond isolated physicochemical descriptors and integrate experimentally derived biological signatures to improve translational prediction at the nano–bio interface [62].

### 3.2. Protein Corona Formation and Biological Identity

Protein corona formation is a critical determinant of nanopharmaceutical behavior in biological environments [60,61]. After exposure to blood, interstitial fluid, or other biological media, nanoparticles rapidly adsorb proteins, lipids, metabolites, and other biomolecules. This adsorption layer can modify the original synthetic identity of the nanocarrier and generate a new biological identity that influences cellular recognition, biodistribution, immune clearance, uptake, toxicity, and therapeutic efficacy [59,60,61,62].

Corona formation is dynamic and context-dependent. Although nanoparticle size, surface charge, hydrophobicity, curvature, material composition, and surface functionalization influence protein adsorption, the final corona also depends on the biological medium, protein concentration, incubation time, disease state, administration route, and species-specific physiology [60,61,68,69]. Therefore, the biological effects of a nanocarrier are often determined not only by its initial formulation properties, but also by the corona that forms after administration.

Traditional experimental approaches have provided valuable information on corona composition, but predicting biological identity remains difficult. Hundreds of biomolecules may compete for the nanoparticle surface, and adsorbed proteins may vary in abundance, orientation, conformation, binding strength, and exchange kinetics [60,61,69]. As a result, simple physicochemical descriptors are usually insufficient to predict corona formation or its biological consequences.

AI offers an important opportunity to model this complexity. Machine learning can integrate nanoparticle descriptors with proteomic datasets to identify adsorption patterns and predict corona composition [62]. These models may reveal how physicochemical properties are associated with the adsorption of opsonins and dysopsonins, and complement proteins, apolipoproteins, immunoglobulins, and albumin [60,61,62,68]. By capturing nonlinear interactions among particle properties and biological media, AI can help classify nanocarriers according to expected biological behavior.

However, AI-driven corona prediction requires careful validation. Proteomic datasets are often generated using different sample preparation methods, incubation conditions, separation techniques, mass spectrometry platforms, and data-processing pipelines [61,62]. Incomplete reporting of nanoparticle synthesis, surface chemistry, batch variability, protein source, incubation time, and dose metrics further limits reproducibility and external validation. These sources of variability may cause models to learn technical artifacts rather than biologically meaningful patterns.

Another limitation is that corona composition alone does not necessarily predict biological outcome. The abundance of a protein in the corona does not define its orientation, receptor accessibility, activation state, or functional consequence [60,61,69]. Therefore, corona prediction models should be linked to functional endpoints such as macrophage uptake, complement activation, cellular internalization, biodistribution, circulation half-life, and toxicity [62,66,67,68].

Overall, AI-assisted corona modeling can support the rational design of nanocarriers with more controlled biological interactions by identifying surface properties associated with reduced opsonization, lower immune recognition, prolonged circulation, or improved targeting [60,61,62,68]. Future studies should prioritize standardized proteomic workflows, dynamic corona modeling, functional validation, uncertainty analysis, and cross-species or patient-specific variability to make protein corona knowledge more useful for translational nanopharmaceutical development.

Practical AI applications in this area have shown that protein corona profiles generated by mass spectrometry can be integrated with supervised learning to predict nanomaterial behavior in vivo. This supports the view that corona composition is not only a descriptive biological feature, but also a predictive signature that can help anticipate nanoparticle recognition, biodistribution, and biological fate under physiologically relevant conditions [62].

### 3.3. Cellular Uptake and Intracellular Trafficking

Cellular uptake and intracellular trafficking are central determinants of nanopharmaceutical efficacy because therapeutic activity depends not only on tissue accumulation, but also on cell entry, intracellular localization, payload release, and the avoidance of premature degradation [59,68,70]. Nanoparticle internalization is influenced by particle size, shape, stiffness, surface charge, hydrophobicity, ligand density, protein corona composition, dose, exposure time, and cell phenotype [68,70,71]. These variables interact with biological mechanisms such as clathrin-mediated endocytosis, caveolae-dependent uptake, macropinocytosis, phagocytosis, and receptor-mediated internalization [70,71].

A major challenge is that uptake is highly context-dependent. The same nanocarrier may show efficient internalization in one cell type but limited uptake in another because of differences in receptor expression, membrane composition, metabolic state, endocytic activity, disease phenotype, or extracellular microenvironment [70,71]. Moreover, high uptake does not necessarily indicate therapeutic efficacy, since nanoparticles may remain trapped in endosomes or lysosomes, undergo degradation, be recycled extracellularly, or fail to release their payload at pharmacologically relevant sites [70,72].

Traditional methods such as flow cytometry, fluorescence microscopy, confocal imaging, and electron microscopy provide valuable information, but they are often semi-quantitative, operator-dependent, or limited to endpoint measurements [35,36,72]. Fluorescence-based assays may also fail to distinguish surface-bound from internalized nanoparticles or capture dynamic trafficking events. These limitations make it difficult to establish robust relationships between nanoparticle design and intracellular behavior.

AI, particularly deep learning, can improve this analysis by processing large microscopy and high-content imaging datasets [35,36,37]. Image-based models can quantify uptake efficiency, segment cellular compartments, classify intracellular localization, detect organelle colocalization, and identify phenotypic changes induced by nanocarrier exposure [35,36]. Convolutional neural networks can extract morphological and spatial patterns that are difficult to identify manually, while temporal models can support the analysis of time-dependent uptake and intracellular transport [35,38].

The integration of AI with high-content imaging allows simultaneous evaluation of nanoparticle distribution, cell morphology, viability, oxidative stress, membrane integrity, inflammatory markers, and organelle localization [37,72]. This multiparametric approach can help distinguish formulations that are merely internalized from those that achieve productive intracellular delivery. Machine learning can also link nanoparticle descriptors, protein corona profiles, ligand characteristics, and imaging-derived cellular features to uptake and trafficking behavior across different cell types [35,37,68].

Despite these advantages, AI-based uptake analysis requires careful validation. Imaging datasets are sensitive to labeling strategy, fluorescence stability, microscopy settings, exposure time, cell density, segmentation method, and nanoparticle concentration [35,36,72]. If these factors are not standardized or included in the model, AI may learn technical artifacts rather than biologically meaningful patterns. Models trained on one cell line or imaging platform may also fail to generalize to other biological systems.

Overall, AI-assisted analysis of cellular uptake and intracellular trafficking can transform complex imaging datasets into quantitative and functionally relevant information. Future studies should move beyond simple uptake quantification and integrate AI-based imaging with mechanistic assays and functional endpoints such as endosomal escape, intracellular drug release, gene silencing, cytotoxicity, immune activation, and therapeutic response. This validation should include biologically relevant systems such as primary cells, co-cultures, organoids, immune–cell models, and patient-derived samples [35,37,72].

Deep learning and machine learning approaches have practical value in this area because they can transform microscopy and high-content imaging datasets into quantitative descriptors of nanoparticle uptake, intracellular localization, organelle colocalization, and cellular phenotype. When combined with mechanistic assays, these image-based AI workflows can help distinguish simple nanoparticle internalization from functionally productive intracellular delivery, including endosomal escape, intracellular payload release, and therapeutic response [35,36,37,70,71,72].

### 3.4. Biodistribution and Pharmacokinetic Modeling

Biodistribution and pharmacokinetics are critical determinants of nanopharmaceutical efficacy, safety, and translational potential [44,45,63]. Unlike small-molecule drugs, nanocarriers do not behave as freely diffusible chemical entities after administration. Their in vivo fate is influenced by particle size, shape, surface charge, stiffness, composition, protein corona formation, carrier degradation, drug release kinetics, immune recognition, vascular permeability, tissue architecture, and clearance by organs such as the liver, spleen, kidneys, and lungs [60,61,62,63,68,69]. Therefore, pharmacokinetic behavior reflects not only the disposition of the active drug, but also the dynamic behavior of the carrier itself.

Traditional pharmacokinetic models may oversimplify nanoparticle-specific processes such as opsonization, mononuclear phagocyte system uptake, organ sequestration, endothelial transport, tumor accumulation, renal filtration, hepatobiliary clearance, and premature drug release [44,45,63]. This is particularly important because the pharmacokinetics of the encapsulated drug, released drug, and intact nanocarrier may differ substantially. Measuring only total drug concentration can therefore provide an incomplete or misleading representation of nanopharmaceutical behavior [44,45].

AI can improve biodistribution and pharmacokinetic prediction by integrating formulation parameters, physicochemical descriptors, biological variables, imaging data, and experimental pharmacokinetic profiles [44,62,63]. Machine learning models can identify nonlinear relationships linking nanoparticle properties to circulation half-life, organ accumulation, clearance pathways, and systemic exposure. These models may help prioritize nanocarrier designs with improved persistence, reduced off-target accumulation, enhanced tissue selectivity, or more predictable elimination behavior. However, these predictions should be interpreted in relation to quantitative delivery efficiency, because increased target accumulation does not necessarily indicate improved therapeutic index if retention in clearance organs also increases [63,73].

However, AI-based pharmacokinetic modeling requires careful interpretation. Available datasets are often small, heterogeneous, and generated using different protocols or measurement methods [44,45,63]. Some studies quantify total drug, whereas others measure labeled carriers, released drug, fluorescence, radioactivity, or elemental content. If these distinctions are not explicitly encoded, AI models may learn misleading associations. Standardized reporting of dose, administration route, sampling time, analytical method, carrier stability, drug release status, and tissue quantification strategy is therefore essential.

Hybrid PBPK–AI models are especially promising because they combine physiological structure with data-driven flexibility [44,45,63]. PBPK models represent tissue compartments, blood flow, vascular permeability, organ volumes, clearance pathways, and physiological parameters, whereas AI can learn nanoparticle-specific corrections related to corona-mediated recognition, macrophage uptake, carrier degradation, or surface-dependent clearance [44,60,61,62,63]. This combination may improve prediction accuracy, preserve biological interpretability, and support interspecies translation from animal models to humans.

AI-driven models can also support the evaluation of therapeutic index by integrating target-site accumulation with exposure in healthy tissues, immune organs, and clearance organs [44,63,73]. A formulation that increases tumor accumulation but also produces high liver or spleen retention may not improve safety or efficacy. Therefore, biodistribution and pharmacokinetic modeling should incorporate exposure, toxicity, efficacy, and dose–response data within multi-objective translational frameworks.

This point also emphasizes why physicochemical optimization alone is insufficient. AI models that accurately predict particle size, surface charge, or encapsulation efficiency may still fail to predict therapeutic benefit if they do not account for organ accumulation, clearance, drug release in vivo, immune recognition, and toxicity. Therefore, biodistribution and pharmacokinetic models should be integrated earlier into AI-guided formulation development so that candidate selection is based not only on CQAs, but also on exposure, target-site delivery, off-target accumulation, and safety-related outcomes [44,45,63,73].

Overall, AI-driven biodistribution and pharmacokinetic modeling can strengthen nanopharmaceutical design by linking formulation attributes, biological context, and systemic exposure. Future studies should prioritize standardized pharmacokinetic datasets, clear distinction between carrier and drug disposition, hybrid PBPK–AI models, external validation, uncertainty analysis, and clinically relevant translation from animal models to humans [44,45,63].

In pharmacokinetic modeling, AI-assisted physiologically based pharmacokinetic approaches have been developed to predict nanoparticle delivery and biodistribution by integrating nanoparticle descriptors with mechanistic PBPK structures. In these workflows, model predictions can be compared with experimentally measured pharmacokinetic profiles, supporting the use of hybrid AI–PBPK models as screening tools for estimating circulation behavior, tissue accumulation, clearance, and tumor delivery efficiency [44,45,63].

### 3.5. Toxicity and Immunogenicity Prediction

Toxicity and immunogenicity remain major barriers to the clinical translation of nanopharmaceuticals [6,7,8,9,64,65]. Although nanocarriers are often designed to improve drug selectivity and reduce systemic exposure, their nanoscale properties may introduce safety risks not observed with conventional formulations. Particle size, surface charge, morphology, hydrophobicity, material composition, degradation products, surface coatings, ligand density, aggregation state, dose, and administration route can influence oxidative stress, membrane disruption, mitochondrial dysfunction, inflammation, complement activation, cytokine release, hemolysis, genotoxicity, and organ-specific accumulation [64,65].

A key challenge is that nanoparticle toxicity is highly context-dependent. A formulation that appears safe in one cell line or animal model may behave differently in another biological system because of differences in immune status, protein corona composition, tissue distribution, clearance mechanisms, and disease microenvironment [59,60,61,62,64]. Toxicity is also influenced by the encapsulated drug, release kinetics, excipients, impurities, sterilization method, and degradation profile. Therefore, safety prediction requires models that integrate physicochemical descriptors, assay conditions, biological endpoints, and exposure metrics [64,65].

AI can support early toxicity prediction by identifying relationships between nanoparticle descriptors and adverse biological responses [64,65]. Machine learning models can estimate risks related to cytotoxicity, oxidative stress, inflammatory activation, hemolysis, genotoxicity, and organ-specific toxicity. These tools may help identify high-risk formulations earlier, prioritize safer design regions, reduce experimental burden, and support safer-by-design development. However, these predictions should be interpreted as risk-prioritization tools rather than replacements for mechanistic and experimental toxicological evaluation.

AI is also relevant for immunogenicity prediction because nanopharmaceuticals interact extensively with the immune system, especially after intravenous administration. Protein corona formation, complement activation, opsonization, macrophage uptake, cytokine release, and anti-carrier immune responses can affect circulation time, biodistribution, infusion reactions, repeated dosing, safety, and efficacy [60,61,62,66,67]. Predictive models that combine nanoparticle surface properties, corona profiles, complement activation data, cytokine signatures, and immune–cell responses may help identify formulations with lower immunological risk.

Despite these advantages, AI-based toxicity prediction faces important limitations. Available nanotoxicology datasets are often small, fragmented, heterogeneous, and inconsistent in nanoparticle characterization, dose metrics, exposure time, biological models, and assay endpoints [64,65]. Many studies report nominal mass concentration without accounting for particle number, surface area, delivered dose, aggregation state, or sedimentation behavior. In addition, nanoparticle interference with optical absorbance, fluorescence, reagent adsorption, or catalytic surface effects may generate misleading assay results [74,75].

Interpretability is also essential. AI models may identify statistical associations between nanoparticle properties and adverse effects without explaining the underlying mechanisms [64,65]. For translational and regulatory use, models should clarify which design features contribute to toxicity or immunogenicity and under which conditions predictions remain reliable. Explainable AI, feature importance analysis, uncertainty estimation, external validation, assay-interference control, and mechanistic validation are therefore necessary [64,65,74].

Overall, AI-based toxicity and immunogenicity prediction can strengthen nanopharmaceutical development by enabling earlier identification of safety risks and supporting safer-by-design strategies. Future studies should prioritize standardized nanotoxicology datasets, harmonized dose metrics, immune-specific endpoints, complement activation assays, cytokine profiling, macrophage uptake, repeated-dose immunogenicity, and validation across independent biological systems [64,65,66,67,74,75].

For safety- and activity-related prediction, quantitative nanostructure–activity relationship models have demonstrated that nanoparticle biological responses can be modeled from structural and physicochemical descriptors. Such approaches can support early risk prioritization, safer-by-design nanomaterial development, and the identification of formulation attributes associated with adverse biological responses, although their translational value remains dependent on dataset quality, descriptor standardization, applicability-domain definition, and experimental validation [19,64,65,74,75].

### 3.6. Toward Precision Nanomedicine

The integration of artificial intelligence with patient-specific data offers an important opportunity to advance nanopharmaceutical development toward precision nanomedicine [1,13,76]. Conventional nanomedicine strategies often rely on population-level assumptions regarding disease biology, biodistribution, drug response, and toxicity. However, patient-to-patient variability in genetics, proteomic profiles, immune status, tumor microenvironment, vascular permeability, metabolic function, microbiome composition, disease stage, and prior treatment history can strongly influence nanocarrier performance [73,76,77].

AI can support precision nanomedicine by integrating genomic, transcriptomic, proteomic, metabolomic, imaging, pathological, pharmacokinetic, and clinical data [13,16,76]. These datasets can help identify patient subgroups more likely to benefit from specific nanotherapeutic strategies, including ligand-targeted nanoparticles, stimuli-responsive systems, immunomodulatory nanocarriers, and nanoparticle-based combination therapies. In this way, AI shifts nanopharmaceutical development from a formulation-centered strategy toward a patient-context-driven model.

One of the most promising applications is patient stratification. In oncology, for example, tumor perfusion, extracellular matrix density, immune infiltration, receptor expression, hypoxia, and vascular permeability can vary substantially between patients and even within the same tumor [76,77]. These factors affect nanoparticle accumulation, penetration, retention, cellular uptake, and therapeutic response. By integrating imaging biomarkers, molecular profiles, and clinical outcomes, AI may help identify which patients are most likely to respond to a given nanotherapeutic platform [13,16,78].

AI may also contribute to personalized safety assessment. Nanopharmaceutical toxicity and immunogenicity can vary according to immune function, complement activity, liver and kidney function, inflammatory status, comorbidities, and previous exposure to similar carriers or excipients [64,65,66,67]. Models that incorporate clinical laboratory data, immune biomarkers, pharmacogenomic information, and treatment history may help predict patients at higher risk of infusion reactions, immune activation, altered clearance, or organ-specific toxicity.

Despite its potential, AI-driven precision nanomedicine should be viewed as a long-term translational goal rather than a near-term clinical reality. Most current applications remain based on preclinical models, retrospective analyses, small clinical datasets, or indirect biomarker associations that do not adequately capture patient heterogeneity [7,9,76]. Prospective clinical validation is still largely absent, and the generation of multimodal patient datasets combining omics, imaging, immune profiling, pharmacokinetics, treatment history, toxicity, and clinical outcomes remains expensive, logistically complex, and difficult to harmonize across institutions. These limitations make it difficult to demonstrate whether AI-guided nanotherapeutic selection improves patient outcomes beyond conventional clinical stratification. Therefore, future progress will require staged validation strategies, representative patient cohorts, clinically meaningful endpoints, explainability, privacy protection, bias assessment, and prospective evaluation before patient-specific AI systems can be used for routine nanomedicine decision-making [16,78,79].

Practical, regulatory, and ethical challenges must also be addressed. Omics and imaging datasets are high dimensional, costly, and difficult to harmonize across institutions, while clinical data may be incomplete, biased, or influenced by treatment history, healthcare access, and population underrepresentation [16,79]. AI models used to guide nanotherapeutic selection must therefore be explainable, externally validated, monitored over time, and evaluated for bias, privacy, informed consent, data governance, and accountability [16,47,79]. Early pilot studies should initially focus on feasibility rather than definitive clinical decision-making. Suitable designs may include retrospective-to-prospective validation studies using archived clinical and imaging datasets, prospective observational biomarker studies embedded in early-phase nanomedicine trials, small feasibility studies evaluating whether AI-predicted responders show improved pharmacokinetic or safety profiles, and privacy-preserving multicenter studies using federated or distributed learning. Such staged approaches would allow AI-driven precision nanomedicine to be evaluated gradually while protecting patients, controlling bias, and generating evidence of clinical utility.

Overall, AI-driven precision nanomedicine represents a forward-looking direction for nanopharmaceutical development. Its greatest promise lies in linking nanocarrier design, biological heterogeneity, pharmacokinetic behavior, safety risk, and clinical response at the patient level [1,13,76]. Future studies should move from proof-of-concept models toward clinically validated and ethically responsible decision-support systems that demonstrate improved therapeutic outcomes beyond conventional population-based approaches.

Practical implementation of AI-driven precision nanomedicine will require moving from general predictive models toward clinically validated decision-support frameworks that integrate patient-specific omics, imaging, immune, pharmacokinetic, and clinical data. In this context, AI could help identify patients more likely to benefit from specific nanotherapeutic platforms, predict individualized safety risks, and guide treatment selection, but these applications will require prospective validation, representative patient cohorts, explainability, privacy protection, and bias assessment before routine clinical adoption [1,13,16,76,77,78,79].

Overall, AI-based approaches can help connect nanoparticle design with biological identity, cellular uptake, biodistribution, pharmacokinetics, toxicity, immunogenicity, and patient-specific response. This is particularly important because nano–bio interactions are dynamic, context-dependent, and difficult to predict from physicochemical properties alone. However, the practical use of AI in this area requires standardized biological datasets, mechanistically meaningful descriptors, functional validation, uncertainty analysis, and clinically relevant endpoints.

## 4. Translational Challenges and Regulatory Perspectives

Despite rapid progress in AI-driven nanopharmaceutical design, clinical translation remains challenging [6,7,8,9]. AI can support formulation optimization, prediction of critical quality attributes, nano–bio interaction modeling, pharmacokinetic analysis, safety assessment, process control, and patient stratification [13,16,20,47]. However, successful translation requires more than high model performance. It depends on reproducible data, experimentally validated models, scalable manufacturing, regulatory transparency, and clear evidence that AI-guided decisions improve product quality, safety, or therapeutic outcomes [16,47,80].

The translational complexity of AI-enabled nanopharmaceuticals results from the convergence of two challenging fields. Nanopharmaceutical performance depends on sensitive relationships among material composition, physicochemical attributes, manufacturing conditions, biological identity, biodistribution, toxicity, and clinical context [4,6,7,8,9,80]. AI adds further complexity because its predictions depend on dataset quality, descriptor selection, algorithmic assumptions, interpretability, validation strategy, uncertainty estimation, and lifecycle management [16,47,57,58]. Therefore, both the nanocarrier and the computational model must be evaluated as part of an integrated translational framework.

**Figure 5:**
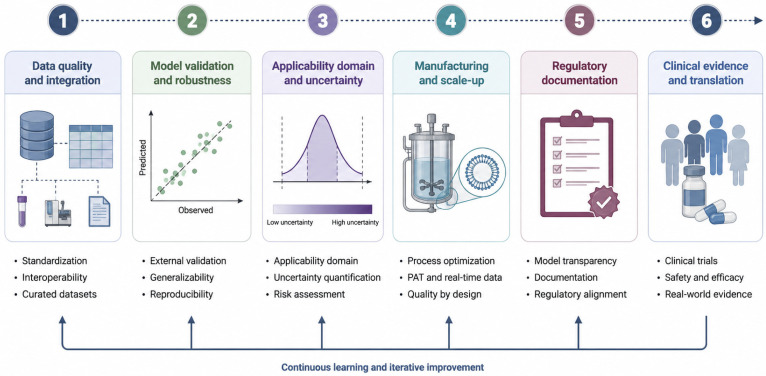
Translational and regulatory roadmap for AI-driven nanopharmaceutical development. The figure summarizes key requirements for translation, including data quality and integration, model validation and robustness, applicability-domain definition and uncertainty quantification, manufacturing scale-up, regulatory documentation, and clinical evidence. Continuous monitoring and iterative improvement are required to maintain model reliability, support lifecycle management, and strengthen regulatory confidence.

As illustrated in Figure 5, AI-driven nanopharmaceutical development should follow a structured pathway linking data generation, model development, preclinical validation, manufacturing scale-up, regulatory assessment, clinical evaluation, and real-world implementation [47,51,57,58,80]. AI may contribute at several stages, including candidate selection, formulation optimization, safety prediction, pharmacokinetic modeling, process monitoring, and post-market surveillance [16,47]. However, the level of evidence required depends on the intended use of the model. Exploratory formulation screening requires less regulatory scrutiny than AI used for release testing, manufacturing control, dose selection, or patient-specific treatment decisions [16,47,78,79].

The figure summarizes the pathway from data generation and model development to validation, manufacturing scale-up, regulatory assessment, and clinical implementation. Cross-cutting requirements include data interoperability, external validation, explainable AI, lifecycle management, interdisciplinary collaboration, and regulatory readiness [16,47,57,58,80].

A primary limitation remains data quality and reproducibility. Nanomedicine datasets are often fragmented, heterogeneous, and difficult to compare across studies because of differences in synthesis methods, raw materials, purification procedures, characterization techniques, biological models, exposure conditions, dose metrics, and reporting standards [20,64,65,81].

If this variability is not properly captured, AI models may learn laboratory-specific patterns rather than generalizable formulation–performance relationships. Standardized reporting of formulation composition, preparation method, batch characteristics, physicochemical properties, biological assay conditions, pharmacokinetic measurements, toxicity endpoints, and experimental metadata is therefore essential [80,81,82].

To make nanopharmaceutical datasets more suitable for AI development, the field should move toward a minimum information standard for AI-ready nanopharmaceutical data. Such a standard should be aligned with FAIR principles and bio–nano minimum reporting recommendations, while also incorporating machine learning-specific requirements [81,82]. At minimum, AI-ready datasets should report nanocarrier composition, raw material source, synthesis or preparation method, processing parameters, batch information, purification procedures, storage conditions, particle size distribution, polydispersity, zeta potential, morphology, surface chemistry, drug loading, encapsulation efficiency, release conditions, stability data, biological model, assay protocol, dose metric, exposure time, pharmacokinetic endpoints, toxicity endpoints, and therapeutic response when available. In addition, datasets should include metadata on units, measurement methods, instruments, preprocessing steps, missing values, replicate structure, experimental variability, and negative or failed formulations. For AI modeling, reported information should also include input descriptors, target variables, training and test set definitions, validation strategy, model performance metrics, uncertainty estimates, applicability domain, and model version. Development of controlled vocabularies, ontologies, and interoperable repositories, following the logic of MIBBI-like minimum information initiatives, would improve dataset comparability, reduce ambiguity, and support external validation across laboratories and nanocarrier platforms.

Practical examples illustrate how these barriers affect AI-enabled nanopharmaceutical development. Machine learning models trained on literature-derived PLGA nanoparticle datasets can predict formulation attributes, but their applicability remains influenced by differences in reported formulation variables, preparation methods, and characterization protocols [17,29,30]. Similarly, AI-assisted pharmacokinetic modeling can improve prediction of nanoparticle delivery and biodistribution, but model performance depends on the availability of comparable pharmacokinetic datasets, clear distinction between carrier and drug disposition, and validation against independent experimental profiles [44,45,63]. These examples show that data heterogeneity and limited external validation are not only methodological concerns, but practical barriers that directly influence model transferability and translational confidence.

Model generalizability and interpretability are equally important. Many AI models are trained on small internal datasets and may fail when applied to different laboratories, nanocarrier platforms, manufacturing scales, animal models, or patient populations [16,20,64,65]. External validation using independent and biologically relevant datasets is therefore essential. In addition, black-box models may be unsuitable when predictions influence product quality, safety, process control, dose selection, or clinical decisions [16,47,79]. Explainable AI, sensitivity analysis, uncertainty quantification, applicability-domain definition, and mechanistic validation are needed to support scientific interpretation and regulatory confidence [16,47,57,58].

The literature examples summarized in Table 2 further emphasize that validation strategies vary substantially across AI applications in nanopharmaceutical research. Some studies rely mainly on retrospective validation using curated or literature-derived datasets, whereas others incorporate direct experimental feedback loops, protein corona-based biological validation, or comparison with measured pharmacokinetic profiles. This variability highlights the need to clearly distinguish exploratory AI models from experimentally validated and translationally relevant predictive frameworks [19,26,37,44,62].

A qualitative assessment of the representative studies discussed in Table 2 indicates that robust external validation remains uncommon. Among these selected examples, approximately three of five studies incorporated validation beyond retrospective or internal prediction, including experimental feedback loops, in vivo biological validation, or comparison with independent pharmacokinetic profiles [37,44,62]. In contrast, formulation- or descriptor-based models still often rely on literature-derived datasets, internal validation, curated datasets, or graphical comparison, with limited evidence of formal cross-laboratory testing or prospective validation [19,26,33]. Although this estimate is not intended as a systematic meta-analysis, it supports the conclusion that external validation and cross-platform testing remain insufficiently reported in AI-driven nanopharmaceutical research.

To improve validation consistency, a tiered validation framework should be adopted. Tier 1 should include internal validation, such as cross-validation, train–test splitting, uncertainty estimation, and applicability-domain definition. Tier 2 should include external validation using independent datasets generated from different experimental batches, laboratories, nanocarrier platforms, or biological models. Tier 3 should include prospective experimental validation, in which AI-predicted formulations or biological outcomes are tested in newly generated experiments. Tier 4 should include translational validation, involving manufacturing-scale assessment, pharmacokinetic or toxicological confirmation, clinically relevant models, and, when appropriate, real-world or clinical evidence. This tiered approach would help distinguish exploratory models from externally validated and translationally actionable AI tools.

Applicability domain is a central concept for the responsible deployment of supervised learning models in nanopharmaceutical development. A model is mathematically reliable only within the descriptor space, formulation range, experimental conditions, nanocarrier classes, and biological contexts represented in its training data. When predictions are made for formulations, materials, process conditions, or biological systems outside this domain, the model is no longer interpolating within learned relationships but extrapolating beyond the evidence used to train it. In such cases, high predictive accuracy during internal validation does not guarantee validity, and predictions may become unreliable, overconfident, or scientifically misleading. Therefore, AI models used in nanomedicine should explicitly define their applicability domain, identify out-of-domain inputs, report uncertainty, and require additional experimental validation before being used for formulation selection, process control, biological prediction, or regulatory decision-making.

### 4.1. Validation, Uncertainty Quantification, and Regulatory Relevance

Uncertainty quantification is essential for translating AI models from exploratory prediction to decision-support tools in nanopharmaceutical development. Without uncertainty estimates, models may provide overconfident predictions for formulations, nanocarrier platforms, biological systems, or manufacturing conditions that differ substantially from the training data. This is particularly risky when predictions are used to guide formulation selection, process adjustment, pharmacokinetic extrapolation, toxicity prioritization, or regulatory decision-making.

Several practical approaches can be used to estimate and communicate uncertainty. Bayesian models and Bayesian neural networks can represent uncertainty in model parameters and predictions, which is useful when datasets are small or heterogeneous. Ensemble methods can estimate uncertainty by comparing predictions from multiple independently trained models, where high prediction variance may indicate limited confidence. Monte Carlo dropout can provide approximate uncertainty estimates in deep learning models by generating repeated predictions under stochastic dropout conditions. Gaussian process models are useful in Bayesian optimization because they provide both predicted responses and uncertainty estimates across the formulation design space. Conformal prediction can generate prediction intervals with defined coverage assumptions, making it useful for communicating whether a new formulation falls within a reliable predictive range. In addition, applicability-domain analysis can identify when a formulation, biological system, or process condition is too different from the training data to support reliable prediction.

For regulatory and translational use, uncertainty quantification should be reported together with model performance metrics, validation strategy, and intended use. Models intended for early formulation screening may tolerate higher uncertainty if predictions are used only to prioritize experiments. In contrast, models used for process control, quality decisions, pharmacokinetic extrapolation, safety assessment, or patient-specific recommendations require stricter uncertainty reporting, predefined acceptance criteria, and revalidation procedures. Therefore, uncertainty quantification should be treated as a core component of AI model documentation, lifecycle management, risk assessment, and regulatory readiness in nanopharmaceutical development [16,47,57,58].

The gap between predicted CQAs and in vivo performance also has regulatory implications. For AI-guided nanopharmaceutical development, a model that predicts particle size, zeta potential, or encapsulation efficiency with high accuracy may still be insufficient for translational decision-making if it is not connected to biological relevance, pharmacokinetics, safety, or therapeutic outcome. Therefore, regulatory-oriented AI workflows should define the intended use of each model and clarify whether it supports early formulation screening, process control, biological prediction, or clinical decision-making [16,47,57,58,80].

### 4.2. Manufacturing, Regulatory, and Ethical Implementation

Manufacturing scalability remains another major barrier. AI-optimized formulations developed at laboratory scale may not retain the same critical quality attributes during pilot or industrial production because nanoparticle formation is highly sensitive to mixing dynamics, flow behavior, batch size, equipment geometry, temperature control, filtration, sterilization, solvent exchange, and raw material variability [12,47,51,80]. Therefore, AI-driven formulation optimization must be connected to quality-by-design, process analytical technology, digital twins, and manufacturing-scale datasets to support reproducible and scalable production [10,12,47,48,49,50,51,52].

Regulatory alignment requires clear documentation and lifecycle management. AI models used in nanopharmaceutical development should have defined objectives, input variables, training data, preprocessing methods, algorithm selection, validation strategy, performance metrics, uncertainty analysis, applicability domain, and procedures for model updating [16,47,57,58]. This is particularly important for adaptive models that evolve as new process, manufacturing, or clinical data become available. Version control, revalidation procedures, audit trails, performance monitoring, and predefined operating boundaries are necessary to avoid regulatory uncertainty [47,58,79].

The regulatory landscape for AI-enabled health technologies is also evolving. The FDA Digital Health Software Precertification Pilot Program, although completed, emphasized organization-level excellence, real-world performance monitoring, and total product lifecycle thinking for software-based health technologies. More recent FDA guidance on predetermined change control plans for AI-enabled device software highlights the need to define, validate, and control planned model modifications before implementation. Similarly, the EMA reflection paper on the use of AI in the medicinal product lifecycle emphasizes risk-based oversight, data quality, transparency, validation, human accountability, and compliance across drug discovery, manufacturing, clinical development, pharmacovigilance, and post-authorization activities. For AI-driven nanopharmaceuticals, these developments suggest that self-learning or continuously updated models will require predefined update procedures, performance monitoring, change-control strategies, revalidation triggers, audit trails, and clear assignment of responsibility before they can be accepted in regulated environments [16,17,47,58,79].

Preclinical-to-clinical translation also remains difficult because in vitro and animal models often fail to reproduce human biological complexity [6,7,8,9,76,80]. Differences in protein corona composition, immune response, vascular physiology, tumor microenvironment, organ clearance, and dose scaling can limit the predictive value of preclinical datasets [59,60,61,62,63,73,76]. AI can help identify translational patterns, but it cannot compensate for biologically inadequate experimental systems. Humanized models, organ-on-chip systems, patient-derived samples, prospective validation, and clinically meaningful endpoints should therefore be incorporated into AI-enabled nanopharmaceutical development [78,80,83].

Ethical and data governance issues are also central to the clinical implementation of AI-driven nanopharmaceuticals, particularly in precision nanomedicine. Models that integrate patient-specific omics, imaging, immune, pharmacokinetic, or clinical data require robust informed consent procedures, privacy protection, secure data sharing, and transparent governance structures. Bias prevention is also essential, because models trained on non-representative datasets may produce less reliable predictions for underrepresented patient populations. Therefore, responsible implementation will require interdisciplinary collaboration among formulation scientists, data scientists, clinicians, regulators, ethicists, and social scientists to ensure that AI-supported nanopharmaceutical development remains scientifically valid, clinically useful, equitable, and accountable [16,79].

Several practical measures can help reduce these translational barriers. First, AI studies should report nanoparticle composition, synthesis method, batch characteristics, physicochemical descriptors, biological assay conditions, dose metrics, and experimental metadata in a standardized manner. Second, models should be evaluated using external datasets whenever possible, rather than relying only on internal cross-validation. Third, uncertainty analysis and applicability-domain assessment should be reported to clarify when model predictions are reliable. Fourth, explainable AI tools should be used to identify formulation or biological variables that drive model outputs, followed by experimental confirmation. Finally, models intended for manufacturing, regulatory, or clinical decision-making should include lifecycle management plans, version control, audit trails, predefined revalidation criteria, and transparent documentation of model updates [16,47,57,58,80,81,82].

Overall, the translation of AI-driven nanopharmaceuticals requires a shift from proof-of-concept modeling toward validated, interpretable, and regulatory-ready development frameworks. Reliable implementation depends on standardized datasets, transparent reporting, external validation, uncertainty quantification, interpretable outputs, scalable manufacturing, lifecycle management, ethical governance, and measurable value for patients, clinicians, and regulators. To reinforce the main translational and oversight issues discussed in this section, Table 5 summarizes the key challenges, their impacts, recommended strategies, translational relevance, and representative references [16,47,79,80].

Together, these challenges show that regulatory readiness for AI-driven nanopharmaceuticals depends on the simultaneous control of data quality, external validation, uncertainty quantification, interpretability, manufacturing scalability, lifecycle management, ethical governance, and regulatory documentation. Therefore, translational success will require coordinated efforts among formulation scientists, data scientists, manufacturers, clinicians, ethicists, and regulatory agencies [16,47,57,58,79,80,81,82].

## 5. Future Perspectives

The integration of artificial intelligence with nanopharmaceutical development is expected to shift the field from empirical experimentation toward predictive, adaptive, and translationally oriented development [13,20,47]. As computational capacity, automation, data availability, and model sophistication continue to advance, AI may become increasingly important for formulation design, process optimization, biological prediction, manufacturing control, and regulatory decision-making [16,47,48]. However, this transition will require movement from proof-of-concept studies toward validated, interpretable, and clinically relevant AI frameworks [16,47,80].

Importantly, AI applications in nanopharmaceutical development should not be viewed as having the same level of maturity. The most practically advanced areas are currently those supported by experimental or preclinical validation, including formulation–property prediction, machine learning-guided nanoparticle optimization, protein corona-based biological fate prediction, and AI-assisted pharmacokinetic modeling. These applications have demonstrated measurable value in predicting formulation attributes, guiding experimental design, improving biological interpretation, or supporting biodistribution analysis. In contrast, fully autonomous closed-loop development, patient-specific digital twins, reinforcement learning-based process control, and clinical decision-support systems for precision nanomedicine remain more exploratory. These approaches are promising, but they require stronger validation, regulatory clarity, prospective evaluation, and evidence of clinical or manufacturing benefit before broad implementation.

One of the most promising future directions is the development of closed-loop systems that combine automated experimentation, real-time data acquisition, predictive modeling, and iterative optimization [53,54,55,84]. In these workflows, experimental outputs are continuously used to update AI models and refine formulation or process parameters. Such systems could reduce development time, minimize material consumption, and improve the identification of robust nanocarrier formulations. To be translationally meaningful, however, closed-loop optimization should incorporate not only particle size or encapsulation efficiency, but also stability, release behavior, toxicity, pharmacokinetics, scalability, and regulatory constraints [47,51,53,54,55].

A practical future scenario would involve an autonomous formulation platform in which a target product profile is first defined, including particle size, drug loading, release behavior, stability, toxicity threshold, and desired biological response. Initial formulations would then be prepared using automated or microfluidic systems, characterized in real time, and evaluated by predictive AI models. Bayesian optimization or active learning would select the next experimental conditions, while a digital twin of the formulation and manufacturing process would simulate how changes in mixing, flow rate, temperature, or composition may affect critical quality attributes. Each experimental cycle would update the model, refine the design space, and prioritize candidates with the best balance of performance, manufacturability, and biological safety. Such a workflow would illustrate how closed-loop experimentation and digital twins could reduce empirical screening, improve process understanding, and accelerate translation from laboratory formulation to scalable nanopharmaceutical production [47,48,49,50,51,52,53,84].

Digital twins represent another important frontier. By creating dynamic virtual representations of formulation processes, manufacturing systems, nanocarrier behavior, or patient-specific physiological environments, digital twins may support predictive simulation and real-time decision-making [48,49,50,51,52]. In manufacturing, they may help predict how process parameters affect critical quality attributes. In biological and clinical contexts, they may support simulation of biodistribution, pharmacokinetics, toxicity risk, tissue accumulation, and therapeutic response [44,45,63]. Nevertheless, digital twins in nanomedicine remain largely conceptual and require stronger validation under real experimental, manufacturing, and clinical conditions [48,49,50,51,52].

Multimodal data integration will also be essential for future AI-enabled nanopharmaceuticals. Next-generation models should combine physicochemical descriptors, process parameters, imaging, spectroscopy, omics, protein corona data, toxicological endpoints, pharmacokinetic profiles, and clinical information [13,20,35,62,64]. Deep learning, graph-based models, transformers, and hybrid mechanistic-AI approaches may help extract relationships from these heterogeneous datasets [35,38,41,42,85]. However, increasing model complexity also increases the risk of opacity, bias, and overfitting. Therefore, predictive performance must be accompanied by explainability, uncertainty quantification, mechanistic plausibility, and external validation [16,47,79,82].

The development of open, interoperable data standards and model repositories will be essential for making AI-driven nanopharmaceutical research more reproducible and comparable. Benchmarking initiatives such as MoleculeNet have demonstrated the value of shared datasets and standardized evaluation frameworks for molecular machine learning, and similar resources are needed for nanopharmaceutical formulations, nano–bio interactions, pharmacokinetics, toxicity, and manufacturing data. Such repositories should include standardized descriptors, metadata, assay conditions, model architectures, training datasets, performance metrics, applicability domains, and version histories. In addition, future AI models should be continuously monitored, updated, and revalidated as new experimental, manufacturing, or clinical data become available, particularly when they are used to support process control, regulatory decisions, or patient-specific treatment strategies [16,47,58,82,85].

From an industrial and regulatory perspective, future progress will depend on stronger alignment among academia, industry, clinicians, and regulatory agencies [16,47,80]. Academic studies often emphasize algorithmic performance, whereas industrial and regulatory settings require reproducibility, robustness, documentation, scalability, and patient safety. Standardized datasets, harmonized reporting criteria, shared benchmarking platforms, and validation protocols will be necessary to make AI models comparable, reproducible, and implementable across development pipelines [80,81,82].

This convergence will require new interdisciplinary skill sets and organizational structures. Future AI-driven nanopharmaceutical programs will need teams capable of combining formulation science, nanomaterial characterization, machine learning, pharmacokinetic modeling, process engineering, regulatory science, clinical trial design, ethics, and data governance. In practice, this may require integrated translational units or cross-functional review boards that evaluate AI models not only for predictive accuracy, but also for biological plausibility, manufacturing feasibility, regulatory acceptability, clinical relevance, bias risk, and patient safety. Such structures will be essential to ensure that AI-supported decisions are scientifically justified, operationally feasible, ethically responsible, and aligned with regulatory expectations [16,47,79,80].

The integration of AI with quality-by-design, process analytical technology, and digital manufacturing will be particularly important for scalable translation [10,12,47,48,49,50,51,52]. Future manufacturing systems may use AI to monitor critical process parameters, predict deviations in product quality, and adjust process conditions in real time. This could reduce batch-to-batch variability, improve process robustness, and support continuous manufacturing [47,51,56]. However, these applications will require validated sensors, reliable data pipelines, clear control strategies, and regulatory guidance for model updates and lifecycle management [47,57,58].

Precision nanomedicine is another important future direction. By integrating patient-specific genomic, proteomic, imaging, immunological, pharmacokinetic, and clinical data, AI may help identify which patients are most likely to benefit from a given nanotherapeutic system [13,76,77]. This could improve patient stratification, guide dose selection, reduce toxicity, and support personalized treatment schedules. However, prospective clinical validation, representative patient cohorts, data privacy, bias assessment, explainability, and clinical accountability will be essential before patient-specific AI systems can be widely implemented [16,78,79].

However, AI-driven precision nanomedicine should advance through staged pilot studies before being considered for routine clinical decision-making. Initial studies could use retrospective clinical, imaging, pharmacokinetic, and biomarker datasets to test whether AI models can identify patient subgroups with different nanotherapeutic responses. Subsequent prospective observational studies could evaluate whether AI-predicted responder profiles are associated with measurable differences in biodistribution, toxicity, pharmacokinetics, or treatment outcomes. More advanced pilot studies may then embed AI-based stratification into early-phase nanomedicine trials, while using privacy-preserving multicenter data-sharing strategies to improve model robustness and reduce bias. This stepwise approach would help determine whether AI-guided precision nanomedicine provides clinical value beyond conventional patient stratification [16,78,79].

Overall, the future of AI-driven nanopharmaceuticals is promising, but its success will depend on scientific rigor rather than technological enthusiasm alone. The field should prioritize standardized and interoperable datasets, externally validated models, explainable algorithms, hybrid mechanistic-AI approaches, clinically relevant endpoints, scalable manufacturing integration, and regulatory-ready documentation [16,47,80,81,82]. If these requirements are met, AI could become a transformative framework linking nanocarrier design, biological prediction, manufacturing control, and precision therapy.

To provide a practical roadmap for the coming decade, Table 6 summarizes the main priorities that should guide future AI-driven nanopharmaceutical development. These priorities emphasize not only algorithmic progress, but also the need for open and interoperable data infrastructures, validated model repositories, lifecycle monitoring, regulatory alignment, ethical governance, and clinically meaningful implementation.

Future studies should also report AI applications as transparent case studies rather than only conceptual frameworks. This includes specifying the dataset source, input descriptors, model type, validation strategy, experimental confirmation, performance metrics, and practical outcome achieved. Such reporting would make it easier to compare AI-based approaches across nanopharmaceutical platforms and determine whether predictive models provide measurable improvements in formulation performance, biological prediction, manufacturing robustness, or translational decision-making [16,47,80,81,82].

Overall, future progress in AI-driven nanopharmaceutical development will depend on moving from conceptual or proof-of-concept studies toward transparent, validated, and implementation-ready workflows. Priority areas include closed-loop experimentation, multimodal data integration, hybrid mechanistic–AI modeling, digital twins, scalable manufacturing, regulatory alignment, and precision nanomedicine. These advances should be accompanied by standardized reporting, prospective validation, ethical governance, and clear evidence that AI improves formulation performance, biological prediction, manufacturing robustness, or clinical decision-making.

## 6. Conclusions

Artificial intelligence is changing how nanopharmaceutical development can be planned, analyzed, and translated. Its main contribution is not only the acceleration of formulation screening, but the possibility of connecting variables that are usually evaluated separately, including nanocarrier composition, process parameters, critical quality attributes, nano–bio interactions, pharmacokinetics, toxicity, manufacturing performance, and clinical relevance.

Across the reviewed literature, the most mature applications are currently those related to formulation–property prediction, experimentally guided nanoparticle optimization, protein corona-based biological fate prediction, and AI-assisted pharmacokinetic modeling. These areas already show how computational models can help organize complex datasets, identify relevant formulation drivers, guide experimental design, and support early translational decisions. In contrast, autonomous closed-loop development, patient-specific digital twins, and AI-guided precision nanomedicine remain longer-term goals that require stronger prospective validation, clinical evidence, and regulatory clarity.

A central message of this review is that predictive accuracy alone is not sufficient for translational impact. AI models used in nanopharmaceutical development must be evaluated in relation to their intended use, dataset quality, descriptor relevance, applicability domain, uncertainty, interpretability, and external validation. Models trained only to predict physicochemical attributes such as particle size, zeta potential, or encapsulation efficiency may support early formulation screening, but they cannot be assumed to predict biological or clinical performance unless they are linked to relevant endpoints such as protein corona formation, cellular uptake, biodistribution, pharmacokinetics, toxicity, immunogenicity, and therapeutic response.

The future value of AI in this field will depend on more rigorous and transparent development practices. Minimum information standards, FAIR-aligned datasets, interoperable repositories, standardized validation strategies, uncertainty quantification, and lifecycle management should become routine components of AI-driven nanopharmaceutical research. Equally important, AI workflows should be integrated with experimental confirmation, mechanistic knowledge, quality-by-design principles, process analytical technology, manufacturing-scale evidence, and regulatory documentation.

In conclusion, AI should be interpreted as a translational framework rather than as an isolated computational tool. Its role is to help bridge formulation science, biological prediction, manufacturing control, and clinical decision-making. For AI-driven nanopharmaceuticals to achieve practical impact, the field must move beyond proof-of-concept modeling toward reproducible, explainable, externally validated, ethically responsible, and clinically meaningful workflows that can support safer, more effective, and more scalable nanomedicine development.

## Figures and Tables

**Table 1:** Summary of representative AI strategies, tools, applications, advantages, and translational limitations in nanopharmaceutical development.

AI Approach	Representative Tools, Platforms, or Models	Main Application	Key Advantage	Main Limitation or Relevance	Representative References
Supervised machine learning	Random forest, support vector machines, artificial neural networks, gradient boosting, XGBoost, LightGBM, scikit-learn, MATLAB machine learning toolboxes	Prediction of size, polydispersity index (PDI), zeta potential, loading, release, and stability	Captures nonlinear formulation–property relationships	Requires curated datasets and external validation	[17,23,36]
Unsupervised learning	Principal component analysis, k-means clustering, hierarchical clustering, self-organizing maps, t-distributed stochastic neighbor embedding (t-SNE), uniform manifold approximation and projection (UMAP)	Clustering, pattern recognition, and dimensionality reduction	Identifies hidden formulation groups and descriptor patterns	Interpretation depends on descriptor quality and biological relevance	[23,24]
Deep learning	Convolutional neural networks, recurrent neural networks, long short-term memory networks, transformer-based models, TensorFlow, Keras, PyTorch	Image, omics, uptake, and toxicity analysis	Extracts complex features from large or unstructured datasets	Requires high data volume, annotation quality, and interpretability	[24,25,28]
Bayesian optimization	Gaussian process-based surrogate models, acquisition functions, Optuna, BoTorch, Bayesian optimization libraries	Adaptive formulation optimization	Reduces experimental burden by guiding informative experiments	Depends on the design space, acquisition function, and surrogate model	[23,26,27]
Evolutionary optimization	Genetic algorithms, non-dominated sorting genetic algorithm II (NSGA-II), particle swarm optimization, evolutionary strategies, multi-objective optimization toolboxes	Multi-objective formulation optimization	Explores large formulation and process spaces	May generate solutions that are mathematically optimal but poorly manufacturable	[20,22]
Reinforcement learning	Q-learning, deep Q-networks, policy-gradient methods, actor–critic models, sequential decision-making frameworks	Sequential optimization and process control	Learns from iterative feedback and supports closed-loop decisions	Still poorly validated in nanopharmaceutical development	[23,25,35]
Physics-informed AI	Physics-informed neural networks, mechanistic-constrained neural networks, diffusion-informed models, differential equation-based learning	Release, transport, pharmacokinetic (PK), and process modeling	Adds mechanistic constraints to data-driven prediction	Requires reliable mechanistic assumptions and parameter estimation	[32,33,34]
Hybrid AI–mechanistic models	Machine learning (ML)-assisted physiologically based pharmacokinetic (PBPK) models, hybrid release models, mechanistic–ML process models, COMSOL Multiphysics, MATLAB/Simulink, gPROMS	Integration with physiologically based pharmacokinetic (PBPK) modeling, quality-by-design (QbD), digital twins, and release models	Improves accuracy, interpretability, and extrapolation capacity	Complex to develop, validate, and maintain across model lifecycles	[32,33,34,35]
Explainable AI	Shapley additive explanations (SHAP), local interpretable model-agnostic explanations (LIME), permutation importance, partial dependence plots, feature ablation, interpretable surrogate models	Feature importance, uncertainty, and model interpretation	Improves transparency and regulatory trust	Explanations require experimental and mechanistic confirmation	[28,35,36]

**Table 2:** Representative literature examples of practical AI applications in nanopharmaceutical development, including validation strategy and reported outcomes.

Application Area	Representative Study	AI-Based Approach	Validation Strategy	Main Outcome or Improvement
Nanocarrier formulation design	Noorain et al. investigated poly(lactic-co-glycolic acid) (PLGA) nanoparticles for antiviral drug delivery [17]	Machine learning models trained using literature-derived PLGA nanoparticle data	Retrospective validation using data extracted from published studies	The models predicted nanoparticle size, PDI, drug loading, and encapsulation efficiency, supporting early formulation screening and reducing reliance on trial-and-error development.
High-throughput formulation optimization	Ortiz–Perez et al. developed a machine learning-guided high-throughput nanoparticle design workflow [37]	Active machine learning combined with microfluidic formulation and high-content imaging	Experimental validation through iterative synthesis and testing of poly(lactic-co-glycolic acid)–polyethylene glycol (PLGA-PEG) nanoparticles	The workflow improved nanoparticle uptake in human breast cancer cells after iterative machine learning-guided optimization.
Nano–bio interactions and biological fate	Lazarovits et al. used supervised learning and mass spectrometry to predict the in vivo fate of nanomaterials [38]	Supervised learning based on protein corona evolution and mass spectrometry data	Experimental workflow combining nanoparticle–protein corona analysis with in vivo fate assessment	The study showed that protein adsorption patterns on nanoparticle surfaces could be used to predict biological fate in vivo.
Pharmacokinetic and biodistribution prediction	Chou et al. developed an AI-assisted physiologically based pharmacokinetic model for nanoparticle tumor delivery [18]	AI-based quantitative structure–activity relationship models integrated with PBPK modeling	Model predictions were compared with experimentally measured pharmacokinetic profiles from different nanoparticle datasets	The AI-assisted PBPK model showed good agreement with experimental pharmacokinetic profiles and supported its potential use as a screening tool for nanoparticle delivery efficiency.
Toxicity- and nanoactivity-related prediction	Fourches et al. developed quantitative nanostructure–activity relationship models for nanomaterials [22]	Quantitative nanostructure–activity relationship modeling using nanoparticle descriptors	Model validation using curated nanostructure–activity datasets and predictive performance assessment	The study demonstrated that nanoparticle biological activity can be modeled from structural and physicochemical descriptors, supporting early safety- and activity-related prediction.

**Table 3:** Integration of AI with optimization and manufacturing frameworks in nanopharmaceutical development.

Framework or Tool	Main Role	AI Contribution	Translational Relevance	Representative References
Design of experiments	Structured evaluation of formulation and process variables	Predicts outcomes across the formulation space	Reduces trial-and-error development	[23,24,33]
Response surface methodology	Modeling variable–response relationships	Complements nonlinear prediction and response optimization	Supports formulation optimization	[23,24]
Quality-by-design	Identification of critical material attributes (CMAs), critical process parameters (CPPs), and critical quality attributes (CQAs)	Links formulation, process, and product performance	Supports robust and regulatory-aligned development	[10,23,24]
Process analytical technology	Real-time process and quality monitoring	Detects deviations and supports adaptive control	Improves process robustness and scale-up	[12,47]
Digital twins	Virtual simulation of formulation or manufacturing systems	Enables model updating and scenario testing	Supports scale-up and predictive troubleshooting	[48,49,50,51,52]
Bayesian optimization	Adaptive selection of experimental conditions	Balances exploration, exploitation, and uncertainty	Enables resource-efficient optimization	[53]
Genetic and evolutionary algorithms	Search of large formulation and process spaces	Identifies optimal trade-offs among competing objectives	Supports multi-objective formulation design	[54]
Reinforcement learning	Sequential formulation or process decisions	Learns optimal actions from iterative feedback	Promising for autonomous development platforms	[55]
Continuous manufacturing	Uninterrupted production with quality monitoring	Supports predictive control and process adjustment	Improves scalability and production consistency	[47,51]
AI–quality-by-design (QbD)–process analytical technology (PAT) systems	Integrated design, monitoring, and control	Connects prediction, real-time data, and adaptive control	Bridges laboratory development and industrial translation	[10,12,47,51]

**Table 4:** AI applications in nano–bio interactions, pharmacokinetics, and safety prediction.

Biological Domain	Main Data Sources	AI Applications	Translational Relevance	Representative References
Protein corona formation	Surface properties, biological fluid composition, proteomics	Predicts corona composition and adsorption patterns	Supports rational surface engineering	[60,61,62]
Cellular uptake	Particle descriptors, ligand density, cell type, imaging data	Predicts uptake efficiency and internalization patterns	Helps optimize targeting strategies	[35,36,37,62]
Intracellular trafficking	High-content imaging, organelle markers, time-resolved microscopy	Classifies subcellular localization and trafficking pathways	Supports intracellular delivery and endosomal escape	[35,36,37]
Biodistribution	Tissue accumulation, imaging signals, administration route	Predicts organ accumulation and off-target distribution	Improves tissue targeting and safety	[44,45,62,63]
Pharmacokinetics	Plasma profiles, release data, carrier stability, clearance pathways	Predicts exposure, half-life, and clearance mechanisms	Supports dose design and translational PK modeling	[44,45,63]
PBPK–AI hybrid modeling	Physiological parameters, organ compartments, nanoparticle descriptors	Combines mechanistic PBPK with machine learning correction	Supports interspecies translation and clinical dose prediction	[44,45,63]
Toxicity prediction	Cytotoxicity, oxidative stress, hemolysis, genotoxicity, organ toxicity	Identifies adverse effects and high-risk formulation attributes	Supports safer-by-design development	[64,65]
Immunogenicity	Complement activation, cytokines, immune–cell uptake, corona data	Predicts immune activation and inflammatory risk	Relevant for intravenous and repeated dosing	[66,67]
Precision nanomedicine	Omics, imaging biomarkers, clinical data, immune status	Predicts response and supports patient stratification	Enables individualized nanotherapeutic strategies	[1,13,62]
Multimodal nano–bio modeling	Integrated physicochemical, imaging, omics, toxicity, and PK datasets	Links nanoscale descriptors with biological outcomes	Connects formulation design with clinical translation	[13,20,35,62,64]

**Table 5:** Summary of translational and oversight challenges in AI-driven nanopharmaceutical development, including impacts, recommended strategies, translational relevance, and representative references.

Challenge	Main Impact	Recommended Strategy	Translational Relevance	Representative References
Data heterogeneity	Reduces model robustness and comparability	Adopt FAIR-aligned minimum information standards, standardized metadata, controlled vocabularies, and interoperable repositories.	Supports reproducible model development	[20,64,65,81,82]
Limited dataset size	Increases overfitting and poor generalization	Develop shared multicenter datasets including successful, negative, and failed formulations with harmonized descriptors and metadata.	Enables external validation	[16,20,64,65,82]
Poor descriptor standardization	Limits formulation–performance modeling	Define an AI-ready nanopharmaceutical dataset checklist covering composition, synthesis, processing, physicochemical descriptors, assay conditions, endpoints, validation design, and model metadata.	Improves evidence quality	[20,80,81]
Lack of external validation	Weakens translational credibility	Validate across independent datasets, platforms, biological systems, and manufacturing scales.	Required for regulatory confidence	[16,20,47,80]
Limited interpretability	Reduces trust in AI-guided decisions	Use explainable AI, sensitivity analysis, feature-importance analysis, and mechanistic validation.	Supports risk assessment	[16,47,57,79]
Uncertainty in predictions	May lead to overconfident decisions	Report uncertainty, confidence, and applicability-domain limits.	Enables risk-based decision-making	[16,47,57,58]
Regulatory uncertainty	Creates ambiguity for validation and accountability	Document model objectives, intended use, validation strategy, performance metrics, uncertainty analysis, and change-control procedures.	Supports approval pathways	[47,57,58,80]
Adaptive model management	Raises concerns about drift and revalidation	Use version control, audit trails, monitoring, drift detection, and revalidation triggers.	Maintains lifecycle compliance	[47,58,79]
Manufacturing scale-up	Limits reproducible industrial translation	Integrate AI with QbD, PAT, digital twins, scale-up data, and process validation.	Supports commercial feasibility	[10,12,47,48,49,50,51,52]
Batch variability	May compromise quality, safety, and efficacy	Use real-time monitoring, predictive control, and feedback-based adjustment of critical process parameters.	Improves quality assurance	[12,47,51,56]
Biological complexity	Limits prediction of PK, toxicity, and efficacy	Integrate omics, imaging, PK, toxicology, immune-response, and patient-derived data.	Improves biological relevance	[59,60,61,62,63,64,65,73]
Preclinical-to-clinical gap	Reduces human predictive value	Use humanized models, organ-on-chip systems, patient-derived samples, and prospective validation.	Supports clinical translation	[6,7,8,9,76,78,80,83]
Ethical and privacy concerns	May limit patient-specific AI use	Apply informed consent, privacy-preserving analytics, bias assessment, transparent governance, and accountability mechanisms.	Important for precision nanomedicine	[16,79]
Limited clinical evidence	Weakens confidence in added value	Conduct prospective studies, real-world benchmarking, post-deployment monitoring, and clinical validation.	Demonstrates clinical utility	[7,8,9,16,78,80]

**Table 6:** Practical roadmap for the next decade of AI-driven nanopharmaceutical.

Priority Area	Current Maturity or Priority	Main Objective	Practical Requirements	Expected Impact
Open and interoperable datasets	High priority; foundational requirement for all AI applications	Enable comparable and reusable AI models across laboratories and platforms	Standardized descriptors, metadata, assay conditions, dose metrics, FAIR data principles, and shared reporting formats	Improved reproducibility, external validation, and cross-platform model transferability
Model repositories and benchmarking	High priority; still underdeveloped for nanopharmaceutical-specific datasets	Support transparent comparison of AI models	Public model repositories, benchmark datasets, version control, performance metrics, and applicability-domain reporting	More reliable model selection and reduced duplication of proof-of-concept studies
Closed-loop experimentation	Emerging; experimentally promising but not yet broadly validated in nanopharmaceutical development	Accelerate formulation optimization through iterative learning	Automated formulation preparation, real-time characterization, Bayesian optimization, active learning, and experimental feedback	Reduced experimental burden and faster identification of robust formulations
Hybrid mechanistic–AI modeling	High near-term priority; relevant for interpretability, extrapolation, and regulatory confidence	Improve interpretability and extrapolation	Integration of mechanistic release, transport, pharmacokinetic, and process models with machine learning	More scientifically defensible predictions and stronger regulatory confidence
Digital twins	Emerging to future-oriented; more mature in pharmaceutical manufacturing than in nanopharmaceutical-specific applications	Simulate formulation behavior and manufacturing processes in real time	Process analytical technology data, mechanistic models, sensor integration, model updating, and scenario testing	Improved scale-up, process control, predictive troubleshooting, and manufacturing robustness
Multimodal biological prediction	Emerging; promising for nano–bio interactions but limited by dataset heterogeneity and biological variability	Connect nanoparticle design with biological outcomes	Integration of physicochemical, omics, imaging, protein corona, toxicity, pharmacokinetic, and clinical datasets	Better prediction of nano–bio interactions, toxicity, biodistribution, and therapeutic response
Regulatory-ready AI workflows	High priority; essential for translation into regulated development environments	Prepare AI models for regulated development environments	Documentation, uncertainty analysis, external validation, audit trails, lifecycle management, and revalidation plans	Clearer regulatory pathways and increased confidence in AI-supported decisions
Ethical and clinical governance	High priority; especially relevant for precision nanomedicine and patient-specific data use	Ensure responsible implementation in precision nanomedicine	Informed consent, privacy protection, bias assessment, patient data governance, explainability, and clinical accountability	More equitable, transparent, and clinically acceptable AI-guided nanomedicine
Interdisciplinary organizational structures	High priority; required to connect technical, clinical, manufacturing, regulatory, and ethical domains	Bridge technical, clinical, manufacturing, and regulatory expertise	Collaboration among formulation scientists, data scientists, clinicians, manufacturers, regulators, ethicists, and patient representatives	Better translation from computational prediction to real-world therapeutic benefit

## Data Availability

No new data were created or analyzed in this study. Data sharing is not applicable to this article.
